# Immune Mechanisms in Cardiovascular Diseases Associated With Viral Infection

**DOI:** 10.3389/fimmu.2020.570681

**Published:** 2020-10-22

**Authors:** Radha Gopal, Michael A. Marinelli, John F. Alcorn

**Affiliations:** Department of Pediatrics, University of Pittsburgh, Pittsburgh, PA, United States

**Keywords:** influenza, heart, immune mechanism, myocardial infarction, atherosclerosis, myocarditis, SARS-CoV-2, COVID-19

## Abstract

Influenza virus infection causes 3–5 million cases of severe illness and 250,000–500,000 deaths worldwide annually. Although pneumonia is the most common complication associated with influenza, there are several reports demonstrating increased risk for cardiovascular diseases. Several clinical case reports, as well as both prospective and retrospective studies, have shown that influenza can trigger cardiovascular events including myocardial infarction (MI), myocarditis, ventricular arrhythmia, and heart failure. A recent study has demonstrated that influenza-infected patients are at highest risk of having MI during the first seven days of diagnosis. Influenza virus infection induces a variety of pro-inflammatory cytokines and chemokines and recruitment of immune cells as part of the host immune response. Understanding the cellular and molecular mechanisms involved in influenza-associated cardiovascular diseases will help to improve treatment plans. This review discusses the direct and indirect effects of influenza virus infection on triggering cardiovascular events. Further, we discussed the similarities and differences in epidemiological and pathogenic mechanisms involved in cardiovascular events associated with coronavirus disease 2019 (COVID-19) compared to influenza infection.

## Introduction

Influenza virus infection causes approximately 36,000 deaths and 200,000 hospitalizations each year in the United States. During influenza epidemics, research focuses on lung disease as the main cause of death. However, epidemiological studies reported significant mortality associated with cardiovascular diseases during influenza virus infection (1, 2). Influenza vaccination reduces cardiovascular events associated with influenza virus infection (3–5). Pandemic outbreaks of novel, highly virulent influenza strains can have an even larger impact on healthcare settings. Pandemics increase both the cardiovascular morbidity and mortality rates compared to those associated with seasonal influenza. During the recent H1N1 influenza pandemic, mortality associated with heart disease was higher in children and young adults than adults (6). Dawood et al. estimated that the 2009 influenza A H1N1 pandemic caused 201,200 respiratory deaths worldwide, with an additional 83,300 deaths associated with cardiovascular complications. 80% of these fatalities were in people younger than 65 (7).

During viral infection, the innate and adaptive immune systems activate a variety of signaling pathways that induce type I (IFN*α*/*β*), type II (IFN*γ*), and type III (IFN*λ*) interferons (IFNs), and a large number of inflammatory cytokines and chemokines (8–11). These IFNs and inflammatory mediators recruit monocytes, neutrophils, and macrophages to the lungs for viral control. However, an excessive influx of innate immune cells and the dysregulated production of inflammatory cytokines results in host-mediated pathological responses during viral infection (8, 11–14).

In the heart, influenza-associated injury can occur either directly by viral entry or indirectly through induction of inflammatory mediators, acute phase proteins, and coagulation factors. Atherosclerosis is a common cause of coronary artery disease (CAD), including MI, stroke, and heart failure. The innate and adaptive immune responses to modified lipids in subendothelial space cause a series of events that result in plaque formation in medium- to large-sized arteries. If the inflammation continues, plaques become vulnerable to rupture, leading to myocardial infarction (MI). A variety of cells, including vascular endothelial cells (VE), macrophages, T cells, and vascular smooth cells play a significant role in atherosclerosis. Understanding the impact of influenza infection on these cells will help to identify therapeutic targets. In this review, we analyzed the direct and indirect effects of influenza infection on these cells in the aspects of atherosclerotic progression, plaque rupture, and thrombosis that subsequently cause acute coronary events. We further discussed the potential mechanism involved in influenza associated myocarditis, ventricular arrhythmia, and heart failure.

COVID-19, caused by severe acute respiratory distress syndrome coronavirus 2 (SARS-CoV-2), has emerged as a global pandemic and has caused significant mortality and morbidity worldwide. SARS-CoV-2 is a highly contagious virus that enters the respiratory epithelium through angiotensin-converting enzyme II (ACE2) receptor and causes pneumonia. The effects of SARS-CoV-2 infection vary from mild asymptomatic infection to lethal disease. Clinical presentation in severely infected patients includes acute respiratory distress syndrome, acute cardiac injury, and secondary illness (13). Studies have shown that COVID-19 patients with one or more underlying conditions, including diabetes, hypertension, and cardiovascular diseases, are more likely to be severely ill (13, 15, 16). COVID-19 also contributes to cardiovascular events such as myocarditis, acute coronary syndrome, cardiomyopathy, and arrhythmias. Influenza virus infection and SARS-CoV-2 infection have similarities in pulmonary immune responses, cellular recruitment, and inflammatory cytokine production. Unlike influenza infection, SARS-CoV-2 causes an abnormal vascular coagulopathy in severely infected COVID-19 patients and multi-systemic inflammatory syndrome with cardiac damage in children. In this review, we discuss the possible mechanisms involved in cardiovascular events associated with COVID-19 in comparison with influenza-associated cardiovascular diseases.

## Association Between Influenza Virus Infection and Cardiovascular Diseases

Several studies have shown that influenza virus infection can trigger detrimental cardiovascular events (3, 15–21). The association between influenza virus infection and non-respiratory causes of death was first identified in the 1930s (22). A case series analysis from 1959 to 1999 showed that mortality from ischemic heart disease (IHD), cerebrovascular disease, and diabetes was highly correlated with influenza and pneumonia cases (23). Another study collected autopsy data between 1993 and 2000 in patients who died from MI and IHD and identified that the odds for MI (1.3, 95% confidence interval (Cl): 1.08–1.56) and chronic IHD (1.10 (95% CI; 0.97–1.26) were increased during influenza seasons (1). A time-series analysis has shown that seasonal influenza virus infection-associated emergency visits correlated with an increase in MI-related mortality, especially in individuals 65 and older (24). Further, a recent study has confirmed that the risk of MI is six times higher during the acute phase (days 1–7) of laboratory-confirmed influenza virus infection (2). These data show the association between influenza infection and MI.

Several groups have analyzed antibody and cellular responses to influenza virus infection in MI patients. Guan et al. found a positive association between IgG and influenza A/B in patients with MI when compared to patients without MI (18, 25). A recent study has shown that influenza-associated cardiovascular excess mortality, including ischemic heart disease, is higher with influenza B virus infection than pandemic influenza A strains (H1N1 and H3N2) (26).

Multiple studies have analyzed whether seasonality impact influenza-associated cardiovascular diseases. A report from central Bohemia has shown that increased influenza epidemics in February positively correlated with a peak in MI incidence (16). A time-series study from 1998 to 2008 has shown that the increase in MI cases associated with influenza virus infection is similar in both temperate and subtropical climates (27).

Studies have also analyzed whether influenza or other respiratory viral infections have a similar impact on triggering cardiovascular diseases. Kwong et al. have shown that the incidence ratio for MI is higher with influenza virus than with respiratory syncytial virus and other viral infections (2). Warren-Gosh et al. (28) have shown that, when compared to other viral infections, influenza has a stronger correlation with triggered MI (28). These studies suggest that the effect of influenza virus infection on triggering cardiovascular events is greater than that of other viral infections.

ST-segment myocardial infarction (STEMI) is a severe condition when the coronary artery is completely blocked in which the patient requires immediate reperfusion therapy and percutaneous coronary intervention (PCI). A non-STEMI (NSTEMI) presentation of MI is due to partial blockage of the coronary artery. Vejpongsa et al., 2019 observed STEMI (9.7%) and NSTEMI (90.3%) cases among MI patients with influenza viral infection. Another study analyzed cardiac injury markers in 143 veterans who were positive for influenza within the previous 30 days showing that 25% of patients had NSTEMI, and 24% had probable STEMI. These studies suggest that both STEMI and NSTEMI presentations of MI are present among influenza-infected MI patients (29).

Influenza infection can trigger myocarditis (29–31), ventricular arrhythmia (32) or heart failure (33). The frequency and damage of myocardium caused by pandemic H1N1 is higher than the seasonal influenza infection (29). ECG reports from H1N1 influenza-infected patients has shown the ventricular dysfunction is associated with influenza infection (34). Another study has shown abnormal ECG findings on days 1, 4, 11, and 28 days after the influenza disease presentation in young adults (35). Some groups also have shown an association between influenza infection and ventricular arrhythmias and hospitalizations of heart failure (32, 33, 36, 37).

Studies have shown elevation of cardiac injury markers and acute phase proteins are indicators of cardiovascular events associated with influenza virus infection (38, 39). Myocardial injury can be determined by serum biomarkers including the MB form of creatine kinase (CK-MB), lactate dehydrogenase (LDH), and troponin (TnT) (40, 41). Cardiac injury markers are shown to be elevated in influenza-positive veterans within 30 days of laboratory confirmation (42). Acute phase proteins are also potential biomarkers for cardiovascular diseases. B-type natriuretic peptide (BNP) and N-terminal proBNP (NT-proBNP) are the biomarkers in diagnosis of heart failure (40, 41). Increased levels of C-reactive protein (CRP) and NT-proBNP, along with increased leukocyte numbers, correlated with mortality rate in elderly patients with 2009 H1N1 infections and cardiovascular diseases (43).

Several clinical reports have shown that influenza vaccination reduces influenza-associated cardiovascular events (4, 36, 44–53). Gwini et al. identified that the influenza vaccine-induced protective effect is greater in those receiving the vaccine before mid-November (49). In another study, Hung et al. found that dual pneumococcal and influenza vaccination reduced respiratory, cardiovascular, and cerebrovascular disease (54). Influenza vaccination has beneficial effects not only against influenza virus infection, but also for other diseases. A study has shown that elderly patients with COPD are protected against acute coronary syndrome if they received influenza vaccination (55). Similarly, influenza vaccination decreased hospitalization rates due to heart failure or acute coronary syndrome in elderly patients with chronic kidney disease (CKD) (56). All these studies suggest a possible link between influenza virus infection, cardiovascular diseases, and a protective role for flu prevention.

## Pathogenic Mechanism Involved in Influenza-Associated Cardiovascular Diseases

### Immune Response to Influenza Virus Infection

Influenza virus enters the lung through airway and alveolar epithelial cells. Viral binding to host cells induces a variety of innate immune signaling, leading to induction of type I and type III IFNs, and pro-inflammatory cytokines (IL-1β, IL-6, and TNFα) and chemokines (CCL2, CCL4, CCL5) (8–11). Type I and type III IFNs bind to their receptors, resulting in activation of Janus kinase (JAK) and signal transducer and activation of transcription (STAT) signaling pathways resulting in the induction of interferon stimulated genes (ISGs), thereby controlling the virus (10, 57, 58).

Pathology during influenza virus infection can be caused by direct viral infection, or indirect damage due to the inflammatory cytokine storm. Influenza virus infection triggers apoptosis or necrosis of alveolar epithelial cells, disrupts tight junction proteins, and damages the endothelium (59–61). Influenza also induces epithelial cell release of a variety of cytokines and chemokines, including TNFα, IL-8, IL-6, CCL2, CCL5, CXCL1, and CXCL10, which attract macrophages and neutrophils to the infection site. These recruited immune cells produce nitric oxide (NO) and reactive oxygen species (ROS) which increase lung injury (62, 63). Further, the inflammatory cytokines may enter the vessel through lung leak or inflammatory cell migration to the circulation (64, 65). Together, these responses increase the accumulation of proteinaceous material in the alveoli, impairing gas exchange and subsequently causing severe respiratory insufficiency (62, 63).

Following the innate immune response, the adaptive immune system plays a role in viral clearance. During influenza virus infection, dendritic cells capture viral antigens and traffic to the draining lymph nodes, presenting antigens to T cells. Antigen presentation occurs on MHC-I and MHC-II molecules to cytotoxic and helper T cells, respectively. Activated effector cytotoxic CD8+ and helper CD4+ T cells migrate from the draining lymph nodes to the lungs and kill viral infected cells. Cytotoxic CD8+ T cells clear the virus or infected cells through induction of IFN*γ*, release of perforin or granzymes, and triggering of apoptosis by Fas/FasL interactions (66–68). CD4+ T cells facilitate IFN*γ* production by CD8+ T cells and virus neutralizing antibody production by B cells (69–72). CD4+ T cells differentiate into Th1, Th2, Th17, T regulatory, or T follicular cells based on the polarizing cytokines produced by dendritic cells. These subsets have specific effects on antiviral responses, promoting B cell responses, and regulation of host immune responses during influenza virus infection.

### Pathophysiology of Atherosclerosis

Atherosclerosis is the most common cause of acute coronary syndrome. A variety of cells, including vascular endothelial cells, macrophages, T cells, and vascular smooth muscle cells are important in atherosclerosis plaque formation. Systemic inflammatory responses, along with direct viral effects on vascular endothelial cells or atherosclerotic plaques during influenza virus infection, may be possible mechanisms in the progression of atherosclerosis or plaque rupture, which can cause subsequent acute coronary events.

The atherosclerotic process is initiated by endothelial dysfunction and accumulation of low-density lipoprotein (LDL) in the sub-endothelial space (73–77). LDL is oxidized (to ox-LDL) by myeloperoxidases and lipoxygenases from immune cells (78). Ox-LDL stimulates the vascular endothelium to increase the expression of adhesion molecules and chemokines that recruit macrophages and T cells into the sub-endothelial space (79–82). Macrophages increase their expression of scavenger receptors, engulf ox-LDL, and become foam cells (83–88). Over time, foam cells undergo apoptosis or necrosis, thus leading to the accumulation of cell debris and the formation of a necrotic core within the intima. Smooth muscle cells then synthesize collagen and elastin to form the fibrous cap that covers the necrotic core. If the fibrous cap is fragile, it may rupture and cause coronary artery disease including MI, stroke, and heart failure (89, 90).

### Direct Effect of Influenza on Atherosclerosis

Influenza-associated effects on atherosclerosis can occur directly by infection of vascular endothelial cells or atherosclerotic plaques, or indirectly through systemic inflammatory responses. Studies have shown the presence of influenza viral antigens in the aorta by PCR and immunohistochemistry (91). Influenza virus has been shown to induce host cell proteases, such as trypsin and matrix metalloprotease 9 (MMP-9), in various organs. This may be a possible mechanism for increased vascular permeability and viral entry in different organs (92). Animal models, including atherosclerotic Apoe^−/−^ mice infected intranasally with influenza virus, have shown antigen localization and influenza viral activity in the aorta (91). However, the cells that potentially carry the virus from the lungs to the aorta are unknown.

Normal vascular endothelial homeostasis is maintained by nitric oxide (NO)-induced relaxing and contracting factors. In normal vascular homeostasis, NO prevents adhesion of leukocytes to the endothelium. In vascular endothelial dysfunction, increased expression of adhesion molecules favors leukocyte binding. It has been shown that HL-60 cells adhere to influenza-infected human umbilical vein endothelial cells (HUVEC) in a viral dose dependent manner (93). Further, adherence depends on the surface hemagglutinin (HA) protein from influenza virus (93).

Systemic and endothelium-induced inflammatory mediators play a role in interrupting endothelial homeostasis. Studies have shown that influenza virus infection increases expression of the chemokines CCL2, CCL5, and IL-8, and the adhesion molecules ICAM1, VCAM-1 and E-selectin in human coronary endothelial cells (HAEC) (91) and increases CXCL10 and CXCL9 in HUVEC cells (94). Another study has shown that, similar to live virus, viral particles also upregulate the expression of chemokine transcripts (95). These data suggest that both whole virus and viral particles contribute to increased antiviral and inflammatory mediators, thereby potentially increasing atherosclerosis.

**Figure 1:**
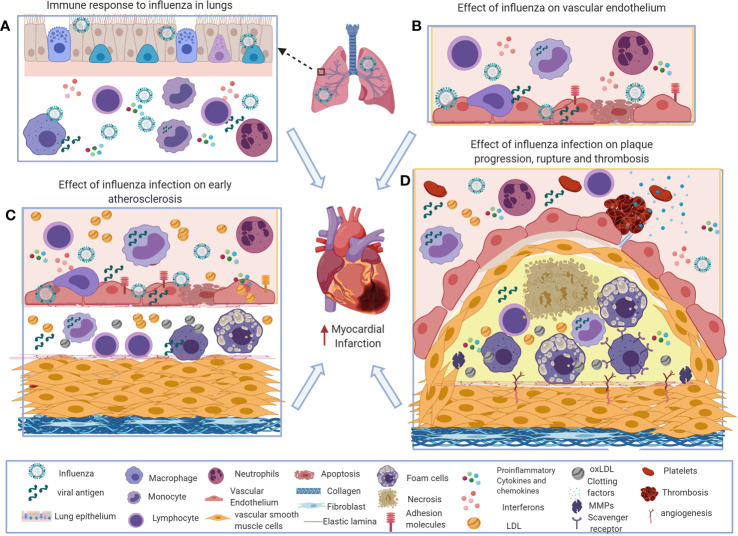
Potential immune mechanisms of influenza-induced exacerbation of atherosclerosis. **(A)** During influenza virus infection, the innate and adaptive immune systems induce interferons and a variety of inflammatory mediators to recruit macrophages, neutrophils, and natural killer (NK) cells to the site of infection to control the virus. Excess influx of innate immune cells and dysregulated production of inflammatory cytokines and chemokines results in pathological responses during influenza virus infection. **(B)** Systemic and local interferons and pro-inflammatory cytokines increase chemotactic factors and adhesion molecules on vascular endothelial cells that increase inflammatory cell recruitment in atherosclerosis. **(C, D)**. Influenza-induced inflammatory mediators increase foam cell formation, activate smooth muscle proliferation, plaque rupture, and thrombosis that exacerbates atherosclerosis and subsequently can cause acute myocardial infarction. This figure was made in ^©^BioRender—biorender.com.

Accumulation of oxidized LDL (ox-LDL) in the sub-endothelial space is the crucial factor in the development of atherosclerosis. Ox-LDL synergistically increases the expression of pro-inflammatory molecules such as IL-1β, IL-6, TNFα, and MMP-9 in response to H1N1 PDM 2009 influenza in HUVEC cells (96). Both influenza and ox-LDL have been shown to increase apoptosis in vascular endothelial cells, the latter through caspase-9 and caspase-3 cascades (87–91). One study has shown that influenza virus infection synergistically increases ox-LDL-induced apoptosis when compared to apoptosis caused by influenza or ox-LDL alone (97). These influenza-induced effects are possible mechanisms involved in atherosclerotic progression (**Figure 1**).

### Indirect Effect of Influenza Virus Infection on Atherosclerosis

The indirect effect of influenza virus infection on atherosclerosis is likely through systemic inflammatory mediators and cell trafficking induced by the virus.

#### Influenza-Induced Inflammatory Mediators and Atherosclerosis

Influenza virus infection induces interferons and a variety of inflammatory cytokines both systemically and locally. Type I, type II, and type III IFNs play an indispensable role in controlling influenza virus (9, 10, 57). In mouse models of atherosclerosis, influenza virus infection increases the expression of ISGs including Mx1 and OAS in the aorta (91). These studies suggest that the influenza viral infection in aorta that induces IFN response.

Studies have shown that type I IFNs promote atherosclerotic plaques by inducing chemotactic factors such as CCL2, CCL3, CCL4, and CCL5, resulting in macrophage accumulation (98), foam cell formation in macrophages (99), and proliferation of smooth muscle cells (100). Type II IFN promotes atherosclerosis by multiple mechanisms (101, 102). IFNγ promotes atherosclerosis by inducing inflammatory mediators such as CCL2, CXCL9, CXCL10, CXCL12, CXCL16, and VCAM1 in vascular endothelial cells, increasing inflammatory cytokines production by macrophages and T cells, promoting foam cell formation by increasing scavenger receptor expression on macrophages, and increasing smooth muscle migration [reviewed in (102)]. Also, IFN*γ* induces MMP production from macrophages and vascular smooth muscle cells, which favors plaque rupture (102).

In addition to interferons, influenza virus infection induces a variety of inflammatory cytokines and chemokines. Pro-inflammatory cytokines (IL-1β, IL-6, and TNFα) have been shown to play a pro-atherogenic role by increasing vascular endothelial adhesion and chemokine production (103). IL-17 promotes plaque formation by either driving type 1 responses or increasing the levels of IL-6, G-CSF, CXCL1, and CCL2 (104–107). In contrast, some studies have shown that IL-17 has a protective role in atherosclerosis by increasing IL-10 (108). Chemokines (CCL2, CCL3 and CCL5) play proatherogenic roles by increasing cellular recruitment and vascular endothelial adhesion [reviewed in (109–112)].

In the Apoe^−/−^ mouse model of atherosclerosis, influenza virus infection increases the levels of IL-1β, IL-6, G-CSF, GM-CSF, CCL2, CCL3, and CCL5 in the serum (91). Also, it has been shown that levels of IL-2, IL-6, IL-18, TNFα, IFN*γ*, ET-1, sICAM-1, and sVCAM-1 are increased in influenza-infected MI patients (25). Collectively, these studies indicate that soluble inflammatory mediators from influenza virus infection may favor atherosclerotic plaque progression (**Figure 1**).

#### Effect of Cellular Trafficking During Influenza Virus Infection on Atherosclerosis

In atherosclerosis, endothelial dysfunction increases adhesion molecules, causing monocytes to migrate into the sub-endothelial space and differentiate into macrophages. Monocytes, especially Ly6C^hi^ monocytes, play an important role in atherosclerotic lesion progression (113, 114). During this process, CCR2, CCR5, and CX3CR1 assist in the recruitment of Ly6C^hi^ monocytes in the lesion (113, 114). Ly6C^hi^ subsets express high levels of CCR2 and resemble inflammatory macrophages, whereas Ly6C^low^ monocytes express high levels of CX3CR1 and resemble tissue repair or resolving-type macrophages. In acute inflammatory conditions, such as MI, increased accumulation of Ly6C^hi^ monocytes accelerates atherosclerosis (115). Ly6C^hi^ monocytes are a key mechanistic player involved in lung pathology during influenza virus infection (116, 117). Due to the high trafficking rates of these cells, it may be possible that Ly6C^hi^ monocytes carry the influenza virus or viral antigen from the lungs to the aorta and favor atherosclerotic lesion progression.

Once the monocytes enter the sub-endothelium, they differentiate into macrophages with the help of growth factors. Further, macrophages increase scavenger receptor expression and engulf ox-LDL becoming foam cells. The local environment is a crucial determinant of the inflammatory (M1) or resolving (M2) macrophage phenotype. IFN*γ* and LPS favor macrophage differentiation into M1 macrophages, while IL-4 drives macrophages towards the M2 phenotype. It has been shown that influenza virus infection in Apoe^−/−^ mice increases macrophage infiltration in the sub-endothelium (118). Another study has shown that influenza virus infection in LDLR^−/−^ mice increases macrophage infiltration into the aortic arch (119). Based on these results, it is possible that an increased proportion of inflammatory (M1) type macrophages favor atherosclerotic lesion progression.

T cells also play a crucial role in the outcome of atherosclerosis. Driven by antigen-specific responses, T cells differentiate into inflammatory effector T (Teff) cells or anti-inflammatory regulatory T (Treg) cells. One study has shown influenza-specific proliferative responses in T cells isolated from atherosclerotic plaques in patients undergoing endarterectomies, suggesting that influenza viral antigens may increase T cell activation and subsequent exacerbation of atherosclerosis (**Figure 1**).

#### Effect of Influenza Virus Infection on Vascular Smooth Muscle Cells

In normal healthy conditions, vascular smooth muscle cells (VSMCs) maintain a contractile or quiescent form and express smooth muscle actin (ACTA2), tangelin (TAGLN), smooth muscle myosin heavy chain (MYH11), and smoothelin markers [reviewed in (86, 120–122)]. In atherosclerosis, inflammatory mediators induced by immune cells and vascular endothelial cells transform these contractile VSMCs into a synthetic or dedifferentiated form. The synthetic form of VSMCs acquires the capacity to proliferate and migrate from media to intima and produce extracellular matrix proteins collagen and elastin, which form the fibrous cap that covers the necrotic core [reviewed in (86, 120–122)]. The transition of VSMC phenotypes may be due to the induction of growth factors, such as platelet derived growth factors, fibroblast growth factors, and matrix metalloproteinases by macrophages and vascular endothelial cells [reviewed in (86, 120–122)]. The dedifferentiated VSMCs can also induce pro-inflammatory cytokines and chemokines (91). In an Apoe^−/−^ mouse model of atherosclerosis, influenza virus infection increased VSMC infiltration into the sub-endothelium (118). Also, an *in vitro* study using human coronary smooth muscle cells has shown that influenza virus infection increases the expression of adhesion molecules (VCAM1 and ICAM1) and production of chemokines (CCL2, CCL5, and IL-8) (91). These studies suggest that influenza virus infection increases smooth muscle cell migration and induction of inflammatory chemokines and adhesion molecules in VSMCs (**Figure 1**).

#### Effect of Influenza Virus Infection on Plaque Rupture

The transition of fatty streak to fibro atheroma occurs with VSMC migration and proliferation (122). The formation of stable or unstable plaques depends on the pro- or anti-inflammatory status of the plaque. Studies have shown that increased levels of IL-4 and IL-10 are associated with stable plaques (123). In contrast, increased levels of IFN*γ* and TNFα are associated with unstable plaques that are highly prone to rupture (102, 124, 125). Increased accumulation of dead macrophages and smooth muscle cells, along with increased matrix degradation products, results in an enlarged necrotic core. These products weaken the fibrous cap, favoring plaque rupture leading to MI (102).

MMPs are known to be among the factors that increase fibrin degradation (MMP1, MMP3, MMP7, MMP9, MMP13, and MMP14) (126–128). An *in vitro* study using HUVEC cells has shown that influenza virus infection increases the expression of MMP9. Another study has shown that the expression of MMP-13 is increased in the atherosclerotic plaques of Apoe^-/-^ mice infected with influenza A virus (129). These studies suggest that influenza virus infection-induced inflammatory mediators may increase plaque destabilization and rupture, leading to MI. One study has shown that the risk of MI is six times higher during the acute phase (days 1–7) of influenza virus infection (2). These results correlate with the excessive inflammatory response during the acute phase of influenza virus infection in the lung, which may increase the chances of plaque rupture and subsequent triggering of MI. Accordingly, a study has shown that influenza vaccination induces stable atherosclerotic lesions in Apoe^−/−^ mice (130). Also, the study showed that influenza vaccination reduces the levels of IFN*γ*, IL-2, and TNFα production and increases the levels of IL-4 in serum (130). These results positively correlate with anti-influenza IgG production from vaccination suggesting that flu prevention by vaccination may limit indirect atherosclerotic damage induced by infection (130) (**Figure 1**).

#### Role of Influenza Virus Infection in Activation of Thrombosis

Plaque rupture releases necrotic components, rich in lipid-laden macrophages, tissue factor, and collagen, into the circulation triggering thrombus formation and leading to acute coronary events. Various mechanisms, including coagulant and anticoagulant factor dysregulation, increased fibrinolysis protease inhibitors, and inflammatory cytokine responses due to vascular infection or injury, increase intravascular coagulation.

The clotting mechanism is initiated once tissue factor and collagen are exposed in the bloodstream and release von Willebrand factor (vWF) [reviewed in (131)]. Tissue factor forms a complex with coagulation factor VII, which in turn activates the extrinsic pathway, whereby collagen release in the blood initiates the intrinsic pathway (131). Activation of both pathways resulting in fibrin deposition and subsequent thrombus formation [reviewed in (131)]. It has been shown that influenza virus infection increases tissue factor and vWF expression in the vascular endothelium (132, 133). Pro-inflammatory cytokines (IL-1β, TNFα, and IL-6) were shown to increase tissue factor in endothelial cells (134). Hypoxia also increases tissue factor expression in the vascular space (135, 136). All these studies suggest that influenza infection directly or indirectly induces clotting factors that may enhance thrombus formation in atherosclerosis.

In contrast, dysregulation of anticoagulant factors such as protein C, antithrombin, and tissue factor pathway inhibitor (TFPI) also enhance thrombus formation. The expression of protein C is activated through a cell surface receptor, thrombomodulin (TM). Studies have shown that influenza virus infection decreases protein C activity, thereby inducing the clotting cascade (137, 138).

Plasminogen activator inhibitor (PAI), a serine protease inhibitor, regulates fibrinolysis and enhances clot formation. Pro-inflammatory cytokines (IL-1β, TNFα, and IL-6) are known to increase PAI-1 activation (139, 140). Influenza virus infection has been shown to increase PAI-1 levels in plasma (137). D-dimer, one of the commonly used markers of fibrin degradation, is also used in the diagnosis of venous thromboembolism. Wang et al. has shown that increases in D-dimer levels correlate with hypoxemia during influenza virus infection (141).

Immunothrombosis, a mechanism involved in the interaction of leukocytes with platelets increases the clot formation. Inflammatory cytokines in the circulation increase receptors on vascular endothelial cells for platelet binding and activation. The activated platelets interact with neutrophils to form neutrophil extracellular traps (NETs) to kill microbes. These neutrophil-platelet aggregates in the circulation may also increase thrombosis (142). Influenza infection has been shown to increase NETs that may favor thrombus formation (143, 144). These studies collectively suggest that influenza virus-induced coagulation factors, fibrinolysis protease inhibitors, and pro-inflammatory immune responses increase thrombosis that subsequently increases the possibility of coronary heart diseases (**Figure 1**).

### Pathogenic Mechanism Involved in Influenza-Associated Myocarditis, Ventricular Arrhythmia, and Heart Failure

Influenza can trigger myocarditis, ventricular arrhythmia and heart failure through systemic and local inflammatory mediators (29, 145–153). Pan et al. have shown induction of trypsin during influenza virus infection as a mechanism to explain the presence of virus in the heart. They have also shown that influenza virus infection up regulates IL-6, IL-1β, TNFα, and MMPs in the myocardium, and trypsin inhibitors alleviate these effects (146). Kotaka et al. have shown macrophage and lymphocytic infiltration in cardiomyocytes (145). A recent study has shown influenza viral replication in cardiomyocytes and purkinje cells in mice (154). Further, Kenny et al. have shown that interferon-induced transmembrane protein-3 (IFITM3) is crucial in controlling influenza viral replication in the heart (155). These studies suggest that influenza-induced pro-inflammatory cytokines and proteases are a possible mechanism in infection-associated myocarditis. Inflammatory cytokines and chemokines, acute phase proteins, and coagulation factors are shown to be possible mechanisms involved in MI that can cause subsequent heart failure and/or ventricular arrhythmias (147–150, 152, 153). All these studies suggest that there is a direct and indirect effect of influenza virus infection on triggering cardiovascular events.

## Comparison of Cardiovascular Conditions Associated With SARS-CoV-2 and Influenza Infection

Several prospective and retrospective analyses have shown the association between influenza and MI. However, information regarding the link between the SARS-CoV-2 infection and MI is limited. A retrospective case series analysis from COVID-19 patients with STEMI during the initial period of pandemics in New York has shown that out of 18 patients, eight patients had an obstructive coronary artery lesion, and ten patients had a non-obstructive myocardial injury (156). Similarly, a study of 28 COVID-19 patients with STEMI from Italy has shown that 39.3% of patients did not show an obstructive lesion (157). Another report of 79 patients with COVID-19 and STEMI from four hospitals from Italy, Lithuania, Spain, and Iraq from February to April 2020 has shown that patients had stent thrombosis, and they were managed with fibrinolytic and PCI therapy (158). These studies suggest that there is an association between COVID-19 patients and MI. However, the sample sizes are small, and the observation period is too short to draw finite conclusions.

Similar to 2009 pandemic H1N1 influenza infection; several cases of myocarditis have been reported in COVID-19 patients. A meta summary analyzed 31 studies with a total of 51 myocarditis cases (159). Out of these, 12 patients were diagnosed based on cardiac magnetic resonance imaging (MRI) or histopathology, and 39 patients were diagnoses based on the inflammatory markers and electrocardiogram (ECG) (159). Also, comparable to 2009 H1N1 influenza infection, several fulminant myocarditis cases were observed in COVID-19 patients (160–168). These data suggest that there are similarities in pandemic H1N1 influenza infection and SARS-CoV-2 infection in triggering myocarditis. However, the incidence of myocarditis due to seasonal influenza is rare and for SARS-CoV-2 unknown.

Unlike influenza infection, SARS-CoV-2 infected children sometimes present with a condition called a multisystemic inflammatory syndrome (MIS-C). In April 2020, the first few cases were observed in the United Kingdom, and later a case in the United States was observed with Kawasaki disease with concurrent COVID-19 (169, 170). Further, a study has shown that 76% of 21 children with Kawasaki disease show evidence of myocarditis (171). Also, a case series analysis of 58 hospitalized children with SARS-CoV-2 has shown 22% with Kawasaki disease and 14% coronary artery dilatations (172). Another systemic review from 39 observational studies from 662 patients showed that the patients presented with gastrointestinal symptoms and abnormal inflammatory, coagulation, cardiac markers, and ECG with decreased heart ejection fraction (173). These data demonstrate aberrant systemic inflammatory responses in children due to MIS-C with COVID-19.

Similar to influenza, studies have shown the association between COVID-19 and cardiac arrhythmias (174–177). Early reports from Wuhan have reported that out of 138 patients, 16.7% patients had arrhythmia, and 44% of these patients were transferred to an intensive care unit (ICU) due to arrhythmia (178). Another study has observed ventricular fibrillation in 5.9% of COVID-19 patients (174). A retrospective case series analysis of five COVID-19 patients with ARDS has shown that ventricular arrhythmia was a primary cause of death of these patients (179). These studies suggest that cardiac arrhythmia is among the most common complications in severely ill COVID-19 patients.

Comparable to influenza infection, reports have also shown heart failure is associated with COVID-19. Zhou et al., 2020 has shown that 23% of 191 COVID-19 patients had heart failure (14). Takotsubo cardiomyopathy, a reversible condition, occurs due to physical and emotional stress that affects the left ventricle. Several reports have shown Takotsubo cardiomyopathy in COVID-19 patients (180–185). Correspondingly, this condition is also reported in influenza-infected patients (186–189). These reports suggest that cardiomyopathy, especially stress-induced cardiomyopathy is reported in influenza viral infected and COVID-19 patients.

Like influenza infection, cardiac injury markers and acute-phase proteins are elevated in critically-ill patients with COVID-19 (174, 190). Shi et al. have shown that out of 416 hospitalized patients, 82 (19.7%) had increased levels of cardiac injury markers, including CRP, procalcitonin, CK-MB, myohemoglobin, troponin (TnT), and N-terminal pro-B-type natriuretic peptide (NTproBNP). Increased mortality rate (51.2%) was observed in patients with cardiac injury when compared to those without cardiac injury (4.5%) (190). Guo et al. demonstrated that in fatal cases of COVID-19, TnT levels rose over time from patient admission to shortly before death (174). Further, TnT levels had a significant correlation with CRP and plasma NTproBNP levels. Cao et al. has shown that 11% of the COVID-19 patients had increased TnT levels, and these patients had no preexisting cardiovascular conditions (191). These data suggest that increased cardiac injury markers are likely due to viral-induced cardiac injury.

Coagulopathy is one of the most concerning sequelae in COVID-19 patients. Several reports have shown mortality associated with pulmonary emboli and venous thrombosis in severely ill COVID-19 patients (192–199). Interestingly, a study by Wichmann et al. described the results of 12 mandatory autopsies of COVID-19, PCR confirmed patients. Of these patients, seven had deep venous thrombosis that was not known before the autopsy (200). Further, four patients died as a result of pulmonary embolism (200). Several studies also have shown changes in coagulation parameters. Increased D-dimer levels, CRP, Factor VIII, vWF, fibrin degradation product (FDP), longer PT, and activated partial thromboplastin time (APTT) was observed in critically ill COVID-19 patients (13, 178, 201, 202). A report has shown 69% patients were positive for venous thromboembolism, and 23% were positive for pulmonary embolism out of 26 COVID-19 patients tested from ICU (202). In a study of autopsy samples from 38 patients who died from COVID-19, 86% showed platelet-fibrin thrombi in small arterial vessels in the lung (203). In influenza case series analysis, fewer number (5.9%) of pulmonary thrombosis and embolism cases were only reported when compared to COVID-19 (204). Another study from autopsy samples from COVID-19 patients and H1N1 patients have shown nine times more numbers of alveolar capillary microthrombi in COVID-19 patients when compared to influenza-infected patients (205). These studies suggest that the COVID-19 associated pulmonary vascular thrombosis is more pronounced when compared to influenza infection and that may be a possible mechanism involved in increased cardiovascular events.

The preceding studies demonstrate cardiovascular events during acute COVID-19 infection. However, a recent report has shown the cardiovascular consequence of COVID-19 after the recovery. A study of 100 convalescent patients 64–92 days after COVID-19 diagnosis by cardiac magnetic resonance imaging showed ongoing myocardial inflammation in 78% of the patients, and 60% out of these patients had no preexisting conditions (142). These data suggest that cardiac inflammation brings long term cardiovascular sequelae. Future studies with a large sample size with various time points after recovery will provide valuable information on the long-term effects of COVID-19 on the heart.

The discussed studies show similarities and dissimilarities between cardiovascular complications associated with influenza and SARS-CoV-2 infection. Cardiovascular conditions such as MI, myocarditis, cardiomyopathy, arrhythmia, and thrombosis are present in both influenza and SARS-CoV-2 infection. However, the multisystemic inflammatory syndrome is only present in SARS-CoV2 infection. The morbidity and mortality rate due to microvascular thrombi and vascular occlusion are high in COVID-19 patients compared to influenza-infected patients. Further, the incidence rate is higher in SARS-CoV-2 infection than the influenza virus infection. There are several possible explanations for the differences in the incidence rates among influenza and SARS-CoV-2 infections. The number of cases analyzed in SARS-CoV-2 is small, and the observation period is short. Most of these data from COVID-19 patients were analyzed from severely ill patients. The available reports are also in a population in the absence of vaccine for SARS-CoV-2 infection versus a population with available vaccines for influenza virus infection. The incidence rate in COVID-19 may change when the analysis is carried out with large sample size and vaccine availability.

## Pathogenic Mechanism of Cardiovascular Events Associated With COVID-19 in Comparison With Influenza Virus Infection

### Direct Effect of SARS-CoV-2 on Vascular Endothelium and Cardiomyocytes

**Figure 2:**
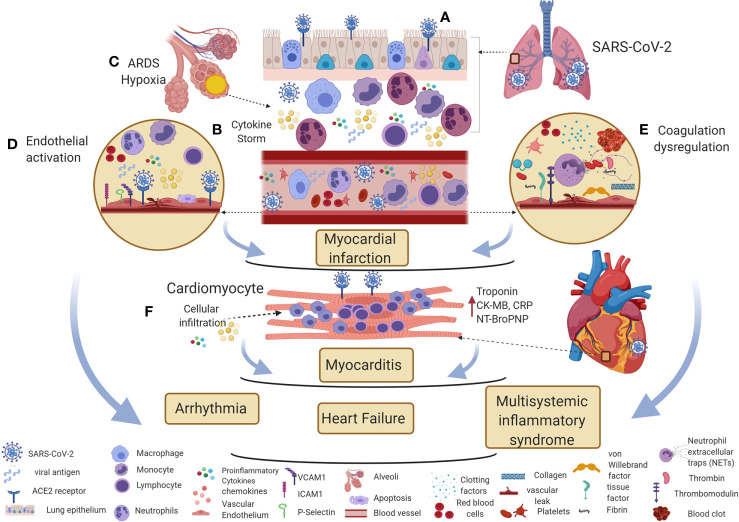
Potential immune mechanisms of COVID-19 associated cardiovascular diseases. **(A)** SARS-CoV-2 enters the respiratory epithelium through the angiotensin-converting enzyme II (ACE2) receptor. The innate immune response induces various cytokines and chemokines to recruit macrophages and neutrophils to control the virus. **(B, C)** The hyperinflammatory cytokine storm increases vascular permeability, decreases gas exchange, stimulates pro-coagulation pathways, and subsequently causes ARDS. **(D, E)** Direct viral entry and inflammatory mediators can activate endothelial adhesion and clotting factors in the vascular space. Inflammatory cytokine storm, oxygen supply/demand mismatch due to hypoxia, endothelial activation, and dysregulation of clotting factors are likely mechanisms involved in triggering myocardial infarction in COVID-19 patients. **(F)** Direct viral entry, along with viral-induced inflammatory mediators increase myocarditis. All these pathological effects lead to arrhythmia, heart failure, and myocardial inflammation in multisystemic inflammatory syndrome. This figure was made in ^©^BioRender—biorender.com.

Like influenza, SARS-CoV-2 may increase risks of cardiovascular events through direct infection or systemic inflammatory responses. A recent report has shown ACE2 receptor expression in the lung, heart, kidney, and gastrointestinal tract (206). The presence of ACE2 receptor in vascular endothelial and VSMCs and myocytes may favor direct viral entry (206, 207). A study has shown viral particles, cellular accumulation, and apoptotic cells in vascular tissue sections from autopsy samples from COVID-19 patients (208). Another report has shown viral RNA in the myocardium in autopsied patients who died from COVID-19. The study also shows increased expression of TNFα, IFN*γ*, CCL-5, IL-6, IL-8, and IL-18 in patients with >1,000 RNA viral copies compared to SARS-CoV-2 negative patients (209). A case report of a child with MIS-C who had cardiac failure demonstrated interstitial and perivascular myocardial cellular infiltration and cardiomyocyte necrosis (210). Further, electron microscopy analysis has shown viral particles in cardiomyocytes and endocardial endothelial cells (210). An *in vitro* study has shown that SARS-CoV-2 virus enters cardiomyocytes and multiplies and transduces a cytopathic effect (211). These reports show the direct effect of the SARS-CoV-2 virus on myocardium and vascular endothelial cells to induce cardiovascular diseases including MI, myocarditis, arrhythmias and heart failure (**Figure 2**).

### Indirect Effect of SARS-CoV-2 Infection on Triggering Cardiovascular Events

In influenza infection, pulmonary induction of type I and type II IFN is a possible mechanism involved in MI. However, in SARS-CoV2 infection, the induction of type I IFN and type III IFNs in respiratory epithelial cells is low (212). The defective IFN responses may lead to an increase in viral multiplication and subsequent increases in inflammatory monocyte accumulation in the lung (213). Also, a study has shown the impaired type I IFN response leads to enhanced pro-inflammatory responses (214). These data suggest that the differences in IFN production and viral control in influenza *versus* SARS-CoV-2 infection may influence the outcome of cardiovascular diseases associated with these viral infections.

During influenza infection, increased production of inflammatory cytokines and increased cellular recruitment may be associated with triggering of cardiovascular diseases. Similarly, Huang et al. have shown increased levels of IL-1β, IL-1RA, IL-7, IL-8, IL-9, IL-10, GCSF, CM-CSF, basic FGF, IFN*γ*, IP-10, MCP-1, MIP-1a, MIP-1b, PDGF, TNFα, and VEGF in COVID-19 infected patients *versus* healthy controls (13). Also, Qin et al. have shown increased levels of TNFα, IL-2R, IL-6 in serum in severe disease compared to mild cases of COVID-19 (215). Studies have shown higher numbers of leukocytes and neutrophils and fewer lymphocytes in the blood of critically ill COVID-19 patients (13, 215). Another study has shown decreased numbers of CD4+ and CD8+ T cells in the blood of severely infected patients than moderately infected patients (216). These dysregulated and hyper-inflammatory cytokine storms may cause increased vascular permeability, decreased gas exchange, activation of pro-coagulation pathways, and subsequently ARDS. Defective gas exchange may increase myocardial injury due to oxygen supply/demand mismatch (217). Like influenza infection, these inflammatory cytokines may trigger cardiovascular diseases such as MI, myocarditis, arrhythmia, and heart failure. Also, these cytokines and dysregulated inflammatory cellular responses may be a possible mechanism in MIS-C in children. These studies suggest that, similar to influenza, cytokine storm induced by SARS-CoV-2 infection is possibly involved in triggering cardiovascular sequelae.

Like influenza, SARS-CoV-2 infection also activates clotting factors, fibrinolysis proteases, and immunothrombosis that favor coagulopathy in COVID-19 patients. A study has shown that platelet-monocyte aggregates were observed in severely ill COVID-19 patients, associated with tissue factor induction (218). SARS-CoV-2 viral entry decreases the expression of ACE2 that enhances the levels of Angiotensin II (Ang II). Further, Ang II is shown to increase tissue factor in monocytes (219). The other major clotting factor, vWF antigen was shown to be increased (mean 565%, SD 199) in ICU and (278%, SD 133) non-ICU COVID-19 patients (220). However, during influenza infection, the levels of vWF antigen (123 to 211%) are lower when compared with COVID-19 patients (221, 222). These studies suggested that induction of the clotting factors is comparatively high in COVID-19 patients *versus* influenza virus infected patients.

Similar to influenza virus infection, reports have shown increased levels of PAI1 in COVID-19 patients. Zhou et al. have shown that the presence of at least 1 µg/ml of D-Dimer is associated with 18 times higher mortality rate in COVID-19 patients (14). Out of 172 COVID-19 patients analyzed, 68% patients showed >0.5 µg/ml of D-Dimer levels suggestive of increased mortality rate among these patients (26). However, in 2009 H1N1 influenza-infected patients, a study has shown concentrations of 1.13 ± 1.09 µg/ml of D-Dimer in patients from non-respiratory failure group *versus* 6.74 ± 5.11 µg/ml in patients in the respiratory failure group (141). Another study has shown levels of D-Dimer from 0.3 to 0.5 µg/ml in seasonal influenza-infected patients. These studies suggest that the D-Dimer level-associated mortality risk is different in influenza virus infected and COVID-19 patients. The differences may be due to the effect of other coagulation factors and inflammatory mediator difference between these two infections.

Studies have also shown that endothelial injury due to SARS-CoV-2 infection increases coagulation marker levels. An autopsy report showed the presence of viral particles and apoptotic bodies in the vascular endothelium (208). Studies have shown elevated expression of endothelial and platelet activation markers ICAM1, VCAM1, P-selectin, sCD40L and thrombomodulin in COVID-19 patients when compared to controls (220, 223). These data suggest that the direct SARS-CoV-2 viral infection also induces procoagulant factors that favor vascular thrombosis. Studies also have shown influenza virus infects vascular endothelial cells, but the thrombotic events are more pronounced in COVID-19 patients.

Several reports have also shown that NET-platelet aggregates favor vascular coagulation in COVID-19 patients. A recent report has shown microvascular thrombi associated with platelet-neutrophil aggregates in the lungs, kidney and the heart (224). Another study has shown NETs in the heart by electron microscopy (EM) in an autopsy sample from COVID-19 patient with MIS-C (210). These studies suggest that similar to influenza infection, immunothrombosis is one of the mechanisms involved in vascular thrombosis in COVID-19 (**Figure 2**).

## Conclusion

Influenza contributes to cardiovascular diseases through a number of different mechanisms. Influenza virus infection increases immune cell recruitment, adhesion, and/or apoptosis, leading to atherosclerotic plaque progression. VSMCs can transform into synthetic VSMCs during the course of infection that then proliferate and migrate into the intima, favoring plaque rupture. The inflammatory response generated during influenza virus infection greatly increases the risks of plaque rupture. Also, influenza-induced coagulation factors may increase thrombosis that can cause acute coronary events. Interactions between influenza-induced pro-inflammatory cytokines and proteases may be a mechanism involved in influenza-induced myocarditis. The highlighted studies illuminate direct and indirect effects of influenza virus infection on triggering or exacerbating cardiovascular diseases.

Based on the presence of ACE2 in various tissues including the lung epithelium, vascular endothelium, and cardiomyocytes, direct SARS-CoV-2 viral-induced effects may trigger cardiovascular events. Several clinical reports from COVID-19 patients show dysregulated production of inflammatory mediators, coagulation factors, and the effect of hypoxia due to ARDS as possible indirect mechanisms involved in triggering cardiovascular events in COVID-19 patients. Epidemiological and pathogenic studies showed similarities and differences between influenza virus and COVID-19 associated cardiovascular diseases. However, the sample sizes analyzed in COVID-19 patients are small, and the observation period is short. The studies are carried out in severely or moderately infected patients. However, identifying the pathogenic mechanisms with the severely ill patients may help to identify therapeutic targets. Animal models will be helpful to understand viral-induced effects on various cells that are involved in triggering cardiovascular events associated with SARS-CoV-2 infection. Potential site and/or cell-specific gene deficient mouse models will help to understand the role of specific cellular responses to pulmonary viral infection on triggering cardiovascular diseases.

## Author Contributions

RG and MM wrote the manuscript. JA provided critical comments and revised the text. All authors contributed to the article and approved the submitted version.

## Funding

The authors are supported by NIH R01 HL107380 (JA) and R01 HL146479 (RG).

## Conflict of Interest

The authors declare that the research was conducted in the absence of any commercial or financial relationships that could be construed as a potential conflict of interest.

## References

[1] Madjid M Miller CC Zarubaev VV Marinich IG Kiselev OI Lobzin YV Influenza epidemics and acute respiratory disease activity are associated with a surge in autopsy-confirmed coronary heart disease death: results from 8 years of autopsies in 34,892 subjects. Eur Heart J (2007) 28(10):1205–10. 10.1093/eurheartj/ehm035 PMC7108465 17440221

[2] Kwong JC Schwartz KL Campitelli MA Chung H Crowcroft NS Karnauchow T Acute Myocardial Infarction after Laboratory-Confirmed Influenza Infection. N Engl J Med (2018) 378(4):345–53. 10.1056/NEJMoa1702090 29365305

[3] Barnes M Heywood AE Mahimbo A Rahman B Newall AT Macintyre CR Acute myocardial infarction and influenza: a meta-analysis of case-control studies. Heart (2015) 101(21):1738–47. 10.1136/heartjnl-2015-307691 PMC4680124 26310262

[4] MacIntyre CR Mahimbo A Moa AM Barnes M Influenza vaccine as a coronary intervention for prevention of myocardial infarction. Heart (2016) 102(24):1953–6. 10.1136/heartjnl-2016-309983 PMC5256393 27686519

[5] Furtado JJ Influenza vaccines for preventing cardiovascular disease. Sao Paulo Med J (2015) 133(4):384. 10.1590/1516-3180.20151334T2 26517151 PMC10876357

[6] Barbandi M Cordero-Reyes A Orrego CM Torre-Amione G Seethamraju H Estep J A case series of reversible acute cardiomyopathy associated with H1N1 influenza infection. Methodist Debakey Cardiovasc J (2012) 8(1):42–5. 10.14797/mdcj-8-1-42 PMC3405785 22891110

[7] Dawood FS Iuliano AD Reed C Meltzer MI Shay DK Cheng PY Estimated global mortality associated with the first 12 months of 2009 pandemic influenza A H1N1 virus circulation: a modelling study. Lancet Infect Dis (2012) 12(9):687–95. 10.1016/S1473-3099(12)70121-4 22738893

[8] Iwasaki A Pillai PS Pillai PS. Innate immunity to influenza virus infection. Nat Rev Immunol (2014) 14(5):315–28. 10.1038/nri3665 PMC4104278 24762827

[9] Durbin RK Kotenko SV Durbin JE Durbin JE. Interferon induction and function at the mucosal surface. Immunol Rev (2013) 255(1):25–39. 10.1111/imr.12101 23947345 PMC5972370

[10] Garcia-Sastre A Durbin RK Zheng H Palese P Gertner R Levy DE The role of interferon in influenza virus tissue tropism. J Virol (1998) 72(11):8550–8. 10.1128/JVI.72.11.8550-8558.1998 PMC110265 9765393

[11] Damjanovic D Small CL Jeyanathan M McCormick S Xing Z Immunopathology in influenza virus infection: uncoupling the friend from foe. Clin Immunol (2012) 144(1):57–69. 10.1016/j.clim.2012.05.005 22673491

[12] Giamarellos-Bourboulis EJ Netea MG Rovina N Akinosoglou K Antoniadou A Complex Immune Dysregulation in COVID-19 Patients with Severe Respiratory Failure. Cell Host Microbe (2020) 27(6):992–1000. 10.1016/j.chom.2020.04.009 32320677 PMC7172841

[13] Huang C Wang Y Li X Ren L Zhao J Hu Y Clinical features of patients infected with 2019 novel coronavirus in Wuhan, China. Lancet (2020) 395(10223):497–506. 10.1016/S0140-6736(20)30183-5 31986264 PMC7159299

[14] Zhou F Yu T Du R Fan G Liu Y Liu Z Clinical course and risk factors for mortality of adult inpatients with COVID-19 in Wuhan, China: a retrospective cohort study. Lancet (2020) 395(10229):1054–62. 10.1016/S0140-6736(20)30566-3 PMC7270627 32171076

[15] Bainton D Jones GR Hole D Influenza and ischaemic heart disease–a possible trigger for acute myocardial infarction? Int J Epidemiol (1978) 7(3):231–9. 10.1093/ije/7.3.231 721358

[16] Dvorakova A Poledne R Influenza–a trigger for acute myocardial infarction. Atherosclerosis (2004) 172(2):391. 10.1016/j.atherosclerosis.2003.09.005 15019551

[17] Finelli L Chaves SS Influenza and acute myocardial infarction. J Infect Dis (2011) 203(12):1701–4. 10.1093/infdis/jir175 21606526

[18] Guan XR Li X Xin XM Jiang LX Cui LY Wang LF Influenza virus infection and risk of acute myocardial infarction. Inflammation (2008) 31(4):266–72. 10.1007/s10753-008-9074-2 18568394

[19] Naghavi M Barlas Z Siadaty S Naguib S Madjid M Casscells W Association of influenza vaccination and reduced risk of recurrent myocardial infarction. Circulation (2000) 102(25):3039–45. 10.1161/01.CIR.102.25.3039 11120692

[20] Hebsur S Vakil E Oetgen WJ Kumar PN Lazarous DF Influenza and coronary artery disease: exploring a clinical association with myocardial infarction and analyzing the utility of vaccination in prevention of myocardial infarction. Rev Cardiovasc Med (2014) 15(2):168–75. 10.3909/ricm0692 25051134

[21] Tillett HE Smith JW Gooch CD Excess deaths attributable to influenza in England and Wales: age at death and certified cause. Int J Epidemiol (1983) 12(3):344–52. 10.1093/ije/12.3.344 6629624

[22] Collins SD Excess Mortality from Causes Other than Influenza and Pneumonia during Influenza Epidemics. Public Health Rep (1896-1970) (1932) 47(46):2159–79. 10.2307/4580606

[23] Reichert TA Simonsen L Sharma A Pardo SA Fedson DS Miller MA Influenza and the winter increase in mortality in the United States, 1959-1999. Am J Epidemiol (2004) 160(5):492–502. 10.1093/aje/kwh227 15321847

[24] Nguyen JL Yang W Ito K Matte TD Shaman J Kinney PL Seasonal Influenza Infections and Cardiovascular Disease Mortality. JAMA Cardiol (2016) 1(3):274–81. 10.1001/jamacardio.2016.0433 PMC5158013 27438105

[25] Guan X Yang W Sun X Wang L Ma B Association of influenza virus infection and inflammatory cytokines with acute myocardial infarction. Inflammation Res (2012) 61(6):591–8. 10.1007/s00011-012-0449-3 22373653

[26] Qi L Li Q Ding XB Gao Y Ling H Liu T Mortality burden from seasonal influenza in Chongqing, China, 2012-2018. Hum Vaccin Immunother (2020) 16(7)1668–735 74. 10.1080/21645515.2019.1693721 PMC7482776 32343618

[27] Warren-Gash C Bhaskaran K Hayward A Leung GM Lo SV Wong CM Circulating influenza virus, climatic factors, and acute myocardial infarction: a time series study in England and Wales and Hong Kong. J Infect Dis (2011) 203(12):1710–8. 10.1093/infdis/jir171 PMC3100509 21606529

[28] Warren-Gash C Hayward AC Hemingway H Denaxas S Thomas SL Timmis AD Influenza infection and risk of acute myocardial infarction in England and Wales: a CALIBER self-controlled case series study. J Infect Dis (2012) 206(11):1652–9. 10.1093/infdis/jis597 PMC3488196 23048170

[29] Ukimura A Satomi H Ooi Y Kanzaki Y Myocarditis Associated with Influenza A H1N1pdm2009. Influenza Res Treat (2012) 2012:351979. 10.1155/2012/351979 23304476 PMC3533457

[30] Joob B Wiwanitkit V To: Fulminant myocarditis associated with the H1N1 influenza virus: case report and literature review. Rev Bras Ter Intensiva (2015) 27(1):82–3. 10.5935/0103-507X.20150014 PMC4396902 25909318

[31] Erden I Erden EC Ozhan H Basar C Acute myocarditis mimicking acute myocardial infarction associated with pandemic 2009 (H1N1) influenza A virus. Cardiol J (2011) 18(5):552–5. 10.5603/CJ.2011.0012 21947992

[32] Madjid M Connolly AT Nabutovsky Y Safavi-Naeini P Razavi M Miller CC Effect of High Influenza Activity on Risk of Ventricular Arrhythmias Requiring Therapy in Patients With Implantable Cardiac Defibrillators and Cardiac Resynchronization Therapy Defibrillators. Am J Cardiol (2019) 124(1):44–50. 10.1016/j.amjcard.2019.04.011 31047651

[33] Kytomaa S Hegde S Claggett B Udell JA Rosamond W Temte J Association of Influenza-like Illness Activity With Hospitalizations for Heart Failure: The Atherosclerosis Risk in Communities Study. JAMA Cardiol (2019) 4(4):363–9. 10.1001/jamacardio.2019.0549 PMC6484790 30916717

[34] Fagnoul D Pasquier P Bodson L Ortiz JA Vincent JL De Backer D Myocardial dysfunction during H1N1 influenza infection. J Crit Care (2013) 28(4):321–7. 10.1016/j.jcrc.2013.01.010 23566732

[35] Ison MG Campbell V Rembold C Dent J Hayden FG Cardiac findings during uncomplicated acute influenza in ambulatory adults. Clin Infect Dis (2005) 40(3):415–22. 10.1086/427282 15668866

[36] Ona MA Bashari DR Tharayil Z Charlot A Hoskins I Timoney M A case of fatal fulminant myocarditis presenting as an acute ST-segment elevation myocardial infarction and persistent ventricular tachyarrhythmia associated with influenza A (H1N1) virus in a previously healthy pregnant woman. Cardiology (2012) 123(2):103–7. 10.1159/000342076 23018755

[37] Saraiya N Singh S Corpuz M Fatal influenza myocarditis with incessant ventricular tachycardia. BMJ Case Rep (2019) 12(7):e228201. 10.1136/bcr-2018-228201 PMC6605955 31266755

[38] Al-Amoodi M Rao K Rao S Brewer JH Magalski A Chhatriwalla AK Fulminant myocarditis due to H1N1 influenza. Circ Heart Fail (2010) 3(3):e7–9. 10.1161/CIRCHEARTFAILURE.110.938506 20484193

[39] Rezkalla SH Kloner RA Influenza-related viral myocarditis. WMJ (2010) 109(4):209–13. 20945722

[40] Nadar SK Shaikh MM Biomarkers in Routine Heart Failure Clinical Care. Card Fail Rev (2019) 5(1):50–6. 10.15420/cfr.2018.27.2 PMC6396063 30847246

[41] Liquori ME Christenson RH Collinson PO Defilippi CR Cardiac biomarkers in heart failure. Clin Biochem (2014) 47(6):327–37. 10.1016/j.clinbiochem.2014.01.032 24530339

[42] Ludwig A Lucero-Obusan C Schirmer P Winston C Holodniy M Acute cardiac injury events </=30 days after laboratory-confirmed influenza virus infection among U.S. veterans, 2010-2012. BMC Cardiovasc Disord (2015) 15:109. 10.1186/s12872-015-0095-0 26423142 PMC4589211

[43] Song BG Wi YM Lee YJ Hong CK Chun WJ Oh JH Clinical features in adult patients with in-hospital cardiovascular events with confirmed 2009 Influenza A (H1N1) virus infection: comparison with those without in-hospital cardiovascular events. J Chin Med Assoc (2012) 75(9):435–41. 10.1016/j.jcma.2012.06.015 22989538

[44] Macintyre CR Heywood AE Kovoor P Ridda I Seale H Tan T Ischaemic heart disease, influenza and influenza vaccination: a prospective case control study. Heart (2013) 99(24):1843–8. 10.1136/heartjnl-2013-304320 PMC3841753 23966030

[45] Mamas MA Fraser D Neyses L Cardiovascular manifestations associated with influenza virus infection. Int J Cardiol (2008) 130(3):304–9. 10.1016/j.ijcard.2008.04.044 18625525

[46] Hsu SY Chen FL Liaw YP Huang JY Nfor ON Chao DY A Matched Influenza Vaccine Strain Was Effective in Reducing the Risk of Acute Myocardial Infarction in Elderly Persons: A Population-Based Study. Med (Baltimore) (2016) 95(10):e2869. 10.1097/MD.0000000000002869 PMC4998863 26962782

[47] Riedmann EM Influenza vaccination reduces risk of heart attack and stroke. Hum Vaccin Immunother (2013) 9(12):2500. 24716204

[48] Phrommintikul A Kuanprasert S Wongcharoen W Kanjanavanit R Chaiwarith R Influenza vaccination reduces cardiovascular events in patients with acute coronary syndrome. Eur Heart J (2011) 32(14):1730–5. 10.1093/eurheartj/ehr004 21289042

[49] Gwini SM Coupland CA Siriwardena AN The effect of influenza vaccination on risk of acute myocardial infarction: self-controlled case-series study. Vaccine (2011) 29(6):1145–9. 10.1016/j.vaccine.2010.12.017 21172383

[50] Siriwardena AN Gwini SM Coupland CA Influenza vaccination, pneumococcal vaccination and risk of acute myocardial infarction: matched case-control study. CMAJ (2010) 182(15):1617–23. 10.1503/cmaj.091891 PMC2952008 20855479

[51] Udell JA Zawi R Bhatt DL Keshtkar-Jahromi M Gaughran F Phrommintikul A Association between influenza vaccination and cardiovascular outcomes in high-risk patients: a meta-analysis. JAMA (2013) 310(16):1711–20. 10.1001/jama.2013.279206 24150467

[52] Liu IF Huang CC Chan WL Huang PH Chung CM Lin SJ Effects of annual influenza vaccination on mortality and hospitalization in elderly patients with ischemic heart disease: a nationwide population-based study. Prev Med (2012) 54(6):431–3. 10.1016/j.ypmed.2012.03.020 22504030

[53] Huang CL Nguyen PA Kuo PL Iqbal U Hsu YH Jian WS Influenza vaccination and reduction in risk of ischemic heart disease among chronic obstructive pulmonary elderly. Comput Methods Programs Biomed (2013) 111(2):507–11. 10.1016/j.cmpb.2013.05.006 23769164

[54] Hung IF Leung AY Chu DW Leung D Cheung T Chan CK Prevention of acute myocardial infarction and stroke among elderly persons by dual pneumococcal and influenza vaccination: a prospective cohort study. Clin Infect Dis (2010) 51(9):1007–16. 10.1086/656587 20887208

[55] Sung LC Chen CI Fang YA Lai CH Hsu YP Cheng TH Influenza vaccination reduces hospitalization for acute coronary syndrome in elderly patients with chronic obstructive pulmonary disease: a population-based cohort study. Vaccine (2014) 32(30):3843–9. 10.1016/j.vaccine.2014.04.064 24837769

[56] Fang YA Chen CI Liu JC Sung LC Influenza Vaccination Reduces Hospitalization for Heart Failure in Elderly Patients with Chronic Kidney Disease: A Population-Based Cohort Study. Acta Cardiol Sin (2016) 32(3):290–8. 10.6515/ACS20150424L PMC4884756 27274169

[57] Durbin JE Fernandez-Sesma A Lee CK Rao TD Frey AB Moran TM Type I IFN modulates innate and specific antiviral immunity. J Immunol (2000) 164(8):4220–8. 10.4049/jimmunol.164.8.4220 10754318

[58] Jewell NA Cline T Mertz SE Smirnov SV Flano E Schindler C Lambda interferon is the predominant interferon induced by influenza A virus infection in vivo. J Virol (2010) 84(21):11515–22. 10.1128/JVI.01703-09 PMC2953143 20739515

[59] Short KR Kasper J van der Aa S Andeweg AC Zaaraoui-Boutahar F Goeijenbier M Influenza virus damages the alveolar barrier by disrupting epithelial cell tight junctions. Eur Respir J (2016) 47(3):954–66. 10.1183/13993003.01282-2015 26743480

[60] Armstrong SM Wang C Tigdi J Si X Dumpit C Charles S Influenza infects lung microvascular endothelium leading to microvascular leak: role of apoptosis and claudin-5. PLoS One (2012) 7(10):e47323. 10.1371/journal.pone.0047323 23115643 PMC3480371

[61] Atkin-Smith GK Duan M Chen W Poon IKH The induction and consequences of Influenza A virus-induced cell death. Cell Death Dis (2018) 9(10):1002. 10.1038/s41419-018-1035-6 30254192 PMC6156503

[62] Cardani A Boulton A Kim TS Braciale TJ Alveolar Macrophages Prevent Lethal Influenza Pneumonia By Inhibiting Infection Of Type-1 Alveolar Epithelial Cells. PLoS Pathog (2017) 13(1):e1006140. 10.1371/journal.ppat.1006140 28085958 PMC5268648

[63] Kalil AC Thomas PG Influenza virus-related critical illness: pathophysiology and epidemiology. Crit Care (2019) 23(1):258. 10.1186/s13054-019-2539-x 31324202 PMC6642581

[64] Hayden FG Fritz R Lobo MC Alvord W Strober W Straus SE Local and systemic cytokine responses during experimental human influenza A virus infection. Relation to symptom formation and host defense. J Clin Invest (1998) 101(3):643–9. 10.1172/JCI1355 PMC508608 9449698

[65] Skoner DP Gentile DA Patel A Doyle WJ Evidence for cytokine mediation of disease expression in adults experimentally infected with influenza A virus. J Infect Dis (1999) 180(1):10–4. 10.1086/314823 10353855

[66] Topham DJ Tripp RA Doherty PC CD8+ T cells clear influenza virus by perforin or Fas-dependent processes. J Immunol (1997) 159(11):5197–200. 9548456

[67] Bot A Bot S Bona CA Protective role of gamma interferon during the recall response to influenza virus. J Virol (1998) 72(8):6637–45. 10.1128/JVI.72.8.6637-6645.1998 PMC109853 9658110

[68] Brincks EL Katewa A Kucaba TA Griffith TS Legge KL CD8 T cells utilize TRAIL to control influenza virus infection. J Immunol (2008) 181(7):4918–25. 10.4049/jimmunol.181.7.4918 PMC2610351 18802095

[69] Allan W Tabi Z Cleary A Doherty PC Cellular events in the lymph node and lung of mice with influenza. Consequences of depleting CD4+ T cells. J Immunol (1990) 144(10):3980–6. 1692070

[70] Bodmer H Obert G Chan S Benoist C Mathis D Environmental modulation of the autonomy of cytotoxic T lymphocytes. Eur J Immunol (1993) 23(7):1649–54. 10.1002/eji.1830230738 8325339

[71] Tripp RA Sarawar SR Doherty PC Characteristics of the influenza virus-specific CD8+ T cell response in mice homozygous for disruption of the H-2lAb gene. J Immunol (1995) 155(6):2955–9. 7673713

[72] Mozdzanowska K Furchner M Maiese K Gerhard W CD4+ T cells are ineffective in clearing a pulmonary infection with influenza type A virus in the absence of B cells. Virology (1997) 239(1):217–25. 10.1006/viro.1997.8882 9426461

[73] Skalen K Gustafsson M Rydberg EK Hulten LM Wiklund O Innerarity TL Subendothelial retention of atherogenic lipoproteins in earlyatherosclerosis. Nature (6890) 2002)417(6890):750–4. 10.1038/nature00804 12066187

[74] Williams KJ Tabas I The response-to-retention hypothesis of early atherogenesis. Arterioscler Thromb Vasc Biol (1995) 15(5):551–61. 10.1161/01.ATV.15.5.551 PMC2924812 7749869

[75] Pentikainen MO Oorni K Ala-Korpela M Kovanen PT Modified LDL - trigger of atherosclerosis and inflammation in the arterial intima. J Intern Med (2000) 247(3):359–70. 10.1046/j.1365-2796.2000.00655.x 10762453

[76] Holvoet P Theilmeier G Shivalkar B Flameng W Collen D LDL hypercholesterolemia is associated with accumulation of oxidized LDL, atherosclerotic plaque growth, and compensatory vessel enlargement in coronary arteries of miniature pigs. Arterioscler Thromb Vasc Biol (1998) 18(3):415–22. 10.1161/01.ATV.18.3.415 9514410

[77] Fukuchi M Watanabe J Kumagai K Baba S Shinozaki T Normal and oxidized low density lipoproteins accumulate deep in physiologically thickened intima of human coronary arteries. Lab Invest (2002) 82(10):1437–47. 10.1097/01.LAB.0000032546.01658.5D 12379778

[78] Navab M Hama SY Reddy ST Ng CJ Van Lenten BJ Laks H Oxidized lipids as mediators of coronary heart disease. Curr Opin Lipidol (2002) 13(4):363–72. 10.1097/00041433-200208000-00003 12151851

[79] Cybulsky MI Gimbrone MA Jr Endothelial expression of a mononuclear leukocyte adhesion molecule during atherogenesis. Science (1991) 251(4995):788–91. 10.1126/science.1990440 1990440

[80] Nakashima Y Raines EW Plump AS Breslow JL Ross R Upregulation of VCAM-1 and ICAM-1 at atherosclerosis-prone sites on the endothelium in the ApoE-deficient mouse. Arterioscler Thromb Vasc Biol (1998) 18(5):842–51. 10.1161/01.ATV.18.5.842 9598845

[81] Khan BV Parthasarathy SS Alexander RW Medford RM Modified low density lipoprotein and its constituents augment cytokine-activated vascular cell adhesion molecule-1 gene expression in human vascular endothelial cells. J Clin Invest (1995) 95(3):1262–70. 10.1172/JCI117776 PMC441465 7533787

[82] Chevre R Gonzalez-Granado JM Megens RT Sreeramkumar V Silvestre-Roig C High-resolution imaging of intravascular atherogenic inflammation in live mice. Circ Res (2014) 114(5):770–9. 10.1161/CIRCRESAHA.114.302590 24366169

[83] Goldstein JL Ho YK Basu SK Brown MS Binding site on macrophages that mediates uptake and degradation of acetylated low density lipoprotein, producing massive cholesterol deposition. Proc Natl Acad Sci U S A (1979) 76(1):333–7. 10.1073/pnas.76.1.333 PMC382933 218198

[84] Suzuki H Kurihara Y Takeya M Kamada N Kataoka M Jishage K A role for macrophage scavenger receptors in atherosclerosis and susceptibility to infection. Nature (1997) 386(6622):292–6. 10.1038/386292a0 9069289

[85] Kunjathoor VV Febbraio M Podrez EA Moore KJ Andersson L Scavenger receptors class A-I/II and CD36 are the principal receptors responsible for the uptake of modified low density lipoprotein leading to lipid loading in macrophages. J Biol Chem (2002) 277(51):49982–8. 10.1074/jbc.M209649200 12376530

[86] Schachter M Vascular smooth muscle cell migration, atherosclerosis, and calcium channel blockers. Int J Cardiol (1997) 62(Suppl 2S85-90). 10.1016/S0167-5273(97)00245-3 9488199

[87] Schwartz SM Smooth muscle migration in atherosclerosis and restenosis. J Clin Invest (1997) 100(11 Suppl):S87–9. 9413408

[88] Schwartz SM Perspectives series: cell adhesion in vascular biology. Smooth muscle migration in atherosclerosis and restenosis. J Clin Invest (1997) 99(12):2814–6. 10.1172/JCI119472 PMC508129 9185501

[89] van der Wal AC Becker AE van der Loos CM Das PK Site of intimal rupture or erosion of thrombosed coronary atherosclerotic plaques is characterized by an inflammatory process irrespective of the dominant plaque morphology. Circulation (1994) 89(1):36–44. 10.1161/01.CIR.89.1.36 8281670

[90] Virmani R Kolodgie FD Burke AP Farb A Schwartz SM Lessons from sudden coronary death: a comprehensive morphological classification scheme for atherosclerotic lesions. Arterioscler Thromb Vasc Biol (2000) 20(5):1262–75. 10.1161/01.ATV.20.5.1262 10807742

[91] Haidari M Wyde PR Litovsky S Vela D Ali M Casscells SW Influenza virus directly infects, inflames, and resides in the arteries of atherosclerotic and normal mice. Atherosclerosis (2010) 208(1):90–6. 10.1016/j.atherosclerosis.2009.07.028 19665123

[92] Wang S Le TQ Kurihara N Chida J Cisse Y Yano M Influenza virus-cytokine-protease cycle in the pathogenesis of vascular hyperpermeability in severe influenza. J Infect Dis (2010) 202(7):991–1001. 10.1086/656044 20731583 PMC7537608

[93] Colden-Stanfield M Ratcliffe D Cramer EB Gallin EK Characterization of influenza virus-induced leukocyte adherence to human umbilical vein endothelial cell monolayers. J Immunol (1993) 151(1):310–21. 7686938

[94] Ishiguro N Takada A Yoshioka M Ma X Kikuta H Kida H Induction of interferon-inducible protein-10 and monokine induced by interferon-gamma from human endothelial cells infected with Influenza A virus. Arch Virol (2004) 149(1):17–34. 10.1007/s00705-003-0208-4 14689273

[95] Wang W Mu X Zhao L Wang J Chu Y Feng X Transcriptional response of human umbilical vein endothelial cell to H9N2 influenza virus infection. Virology (2015) 482117–27. 10.1016/j.virol.2015.03.037 25863179

[96] Wu Y Huang H Synergistic enhancement of matrix metalloproteinase-9 expression and pro-inflammatory cytokines by influenza virus infection and oxidized-LDL treatment in human endothelial cells. Exp Ther Med (2017) 14(5):4579–85. 10.3892/etm.2017.5099 PMC5658766 29104665

[97] Suo J Zhao L Wang J Zhu Z Zhang H Gao R Influenza virus aggravates the ox-LDL-induced apoptosis of human endothelial cells via promoting p53 signaling. J Med Virol (2015) 87(7):1113–23. 10.1002/jmv.24166 25777161

[98] Goossens P Gijbels MJ Zernecke A Eijgelaar W Vergouwe MN van der Made I Myeloid type I interferon signaling promotes atherosclerosis by stimulating macrophage recruitment to lesions. Cell Metab (2010) 12(2):142–53. 10.1016/j.cmet.2010.06.008 20674859

[99] Li J Fu Q Cui H Qu B Pan W Shen N Interferon-alpha priming promotes lipid uptake and macrophage-derived foam cell formation: a novel link between interferon-alpha and atherosclerosis in lupus. Arthritis Rheumatol (2011) 63(2):492–502. 10.1002/art.30165 21280004

[100] Diao Y Mohandas R Lee P Liu Z Sautina L Mu W Effects of Long-Term Type I Interferon on the Arterial Wall and Smooth Muscle Progenitor Cells Differentiation. Arterioscler Thromb Vasc Biol (2016) 36(2):266–73. 10.1161/ATVBAHA.115.306767 26634654

[101] Boshuizen MC de Winther MP Interferons as Essential Modulators of Atherosclerosis. Arterioscler Thromb Vasc Biol (2015) 35(7):1579–88. 10.1161/ATVBAHA.115.305464 25953648

[102] McLaren JE Ramji DP Interferon gamma: a master regulator of atherosclerosis. Cytokine Growth Factor Rev (2009) 20(2):125–35. 10.1016/j.cytogfr.2008.11.003 19041276

[103] Fatkhullina AR Peshkova IO Koltsova EK The Role of Cytokines in the Development of Atherosclerosis. Biochem (Mosc) (2016) 81(11):1358–70. 10.1134/S0006297916110134 PMC5471837 27914461

[104] Smith E Prasad KM Butcher M Dobrian A Kolls JK Ley K Blockade of interleukin-17A results in reduced atherosclerosis in apolipoprotein E-deficient mice. Circulation (2010) 121(15):1746–55. 10.1161/CIRCULATIONAHA.109.924886 PMC2929562 20368519

[105] Ng HP Zhu X Harmon EY Lennartz MR Nagarajan S Reduced Atherosclerosis in apoE-inhibitory FcgammaRIIb-Deficient Mice Is Associated With Increased Anti-Inflammatory Responses by T Cells and Macrophages. Arterioscler Thromb Vasc Biol (2015) 35(5):1101–12. 10.1161/ATVBAHA.115.305290 PMC4409543 25792447

[106] van Es T van Puijvelde GH Ramos OH Segers FM Joosten LA van den Berg WB Attenuated atherosclerosis upon IL-17R signaling disruption in LDLr deficient mice. Biochem Biophys Res Commun (2009) 388(2):261–5. 10.1016/j.bbrc.2009.07.152 19660432

[107] Chen S Crother TR Arditi M Emerging role of IL-17 in atherosclerosis. J Innate Immun (2010) 2(4):325–33. 10.1159/000314626 PMC2895754 20505315

[108] Taleb S Romain M Ramkhelawon B Uyttenhove C Pasterkamp G Herbin O Loss of SOCS3 expression in T cells reveals a regulatory role for interleukin-17 in atherosclerosis. J Exp Med (2009) 206(10):2067–77. 10.1084/jem.20090545 PMC2757872 19737863

[109] Sheikine Y Hansson GK Chemokines and atherosclerosis. Ann Med (2004) 36(2):98–1002 118. 10.1080/07853890310019961 15119830

[110] Mach F The role of chemokines in atherosclerosis. Curr Atheroscler Rep (2001) 3(3):243–51. 10.1007/s11883-001-0067-y 11286646

[111] Reape TJ Groot PH Chemokines and atherosclerosis. Atherosclerosis (1999) 147(2):213–1006, 25. 10.1016/s0021-9150(99)00346-9 10559506

[112] Terkeltaub R Boisvert WA Curtiss LK Chemokines and atherosclerosis. Curr Opin Lipidol (1998) 9(5):397–405. 10.1097/00041433-199810000-00003 9812193

[113] Tacke F Alvarez D Kaplan TJ Jakubzick C Spanbroek R Llodra J Monocyte subsets differentially employ CCR2, CCR5, and CX3CR1 to accumulate within atherosclerotic plaques. J Clin Invest (2007) 117(1):185–94. 10.1172/JCI28549 PMC1716202 17200718

[114] Swirski FK Libby P Aikawa E Alcaide P Luscinskas FW Weissleder R Ly-6Chi monocytes dominate hypercholesterolemia-associated monocytosis and give rise to macrophages in atheromata. J Clin Invest (2007) 117(1):195–205. 10.1172/JCI29950 17200719 PMC1716211

[115] Dutta P Courties G Wei Y Leuschner F Gorbatov R Robbins F Myocardial infarction accelerates atherosclerosis. Nature (2012) 487(7407):325–9. 10.1038/nature11260 PMC3401326 22763456

[116] Lin KL Suzuki Y Nakano H Ramsburg E Gunn MD CCR2+ monocyte-derived dendritic cells and exudate macrophages produce influenza-induced pulmonary immune pathology and mortality. J Immunol (2008) 180(4):2562–72. 10.4049/jimmunol.180.4.2562 18250467

[117] Lin KL Sweeney S Kang BD Ramsburg E Gunn MD CCR2-antagonist prophylaxis reduces pulmonary immune pathology and markedly improves survival during influenza infection. J Immunol (2011) 186(1):508–15. 10.4049/jimmunol.1001002 PMC3723340 21098218

[118] Naghavi M Wyde P Litovsky S Madjid M Akhtar A Naguib S Influenza infection exerts prominent inflammatory and thrombotic effects on the atherosclerotic plaques of apolipoprotein E-deficient mice. Circulation (2003) 107(5):762–8. 10.1161/01.cir.0000048190.68071.2b 12578882

[119] Van Lenten BJ Wagner AC Navab M Anantharamaiah GM Hui EK Nayak DP D-4F, an apolipoprotein A-I mimetic peptide, inhibits the inflammatory response induced by influenza A infection of human type II pneumocytes. Circulation (2004) 110(20):3252–8. 10.1161/01.CIR.0000147232.75456.B3 15533864

[120] Alexander MR Owens GK Epigenetic control of smooth muscle cell differentiation and phenotypic switching in vascular development and disease. Annu Rev Physiol (2012) 74:13–40. 10.1146/annurev-physiol-012110-142315 22017177

[121] Basatemur GL Jorgensen HF Clarke MCH Bennett MR Mallat Z Vascular smooth muscle cells in atherosclerosis. Nat Rev Cardiol (2019) 16(12):727–44. 10.1038/s41569-019-0227-9 31243391

[122] Bennett MR Sinha S Owens GK Vascular Smooth Muscle Cells in Atherosclerosis. Circ Res (2016) 118(4):692–702. 10.1161/CIRCRESAHA.115.306361 26892967 PMC4762053

[123] Tedgui A Mallat Z Cytokines in atherosclerosis: pathogenic and regulatory pathways. Physiol Rev (2006) 86(2):515–81. 10.1152/physrev.00024.2005 16601268

[124] Gupta S Pablo AM Jiang X Wang N Tall AR Schindler C IFN-gamma potentiates atherosclerosis in ApoE knock-out mice. J Clin Invest (1997) 99(11):2752–61. 10.1172/JCI119465 PMC508122 9169506

[125] Zhang L Peppel K Sivashanmugam P Orman ES Brian L Exum ST Expression of tumor necrosis factor receptor-1 in arterial wall cells promotes atherosclerosis. Arterioscler Thromb Vasc Biol (2007) 27(5):1087–94. 10.1161/ATVBAHA.0000261548.49790.63 PMC2522308 17442899

[126] Fic P Zakrocka I Kurzepa J Stepulak A [Matrix metalloproteinases and atherosclerosis]. Postepy Hig Med Dosw (Online) (2011) 65:16–27. 10.5604/17322693.931536 21357991

[127] Beaudeux JL Giral P Bruckert E Foglietti MJ Chapman MJ Matrix metalloproteinases, inflammation and atherosclerosis: therapeutic perspectives. Clin Chem Lab Med (2004) 42(2):121–31. 10.1515/CCLM.2004.024 15061349

[128] Watanabe N Ikeda U Matrix metalloproteinases and atherosclerosis. Curr Atheroscler Rep (2004) 6(2):112–20. 10.1007/s11883-004-0099-1 15023295

[129] Lee HS Noh JY Shin OS Song JY Cheong HJ Kim WJ Matrix Metalloproteinase-13 in Atherosclerotic Plaque Is Increased by Influenza A Virus Infection. J Infect Dis (2020) 221(2):256–66. 10.1093/infdis/jiz580 31693113

[130] Bermudez-Fajardo A Oviedo-Orta E Influenza vaccination promotes stable atherosclerotic plaques in apoE knockout mice. Atherosclerosis (2011) 217(1):97–105. 10.1016/j.atherosclerosis.2011.03.019 21507404

[131] Yang Y Tang H Aberrant coagulation causes a hyper-inflammatory response in severe influenza pneumonia. Cell Mol Immunol (2016) 13(4):432–42. 10.1038/cmi.2016.1 PMC4947825 27041635

[132] Antoniak S Tatsumi K Hisada Y Milner JJ Neidich SD Shaver CM Tissue factor deficiency increases alveolar hemorrhage and death in influenza A virus-infected mice. J Thromb Haemost (2016) 14(6):1238–48. 10.1111/jth.13307 PMC5892427 26947929

[133] Sugiyama MG Gamage A Zyla R Armstrong SM Advani S Advani A Influenza Virus Infection Induces Platelet-Endothelial Adhesion Which Contributes to Lung Injury. J Virol (2016) 90(4):1812–23. 10.1128/JVI.02599-15 PMC4733979 26637453

[134] Szotowski B Antoniak S Poller W Schultheiss HP Rauch U Procoagulant soluble tissue factor is released from endothelial cells in response to inflammatory cytokines. Circ Res (2005) 96(12):1233–9. 10.1161/01.RES.0000171805.24799.fa 15920023

[135] Yan SF Mackman N Kisiel W Stern DM Pinsky DJ Hypoxia/Hypoxemia-Induced activation of the procoagulant pathways and the pathogenesis of ischemia-associated thrombosis. Arterioscler Thromb Vasc Biol (1999) 19(9):2029–35. 10.1161/01.atv.19.9.2029 10479642

[136] Lawson CA Yan SD Yan SF Liao H Zhou YS Sobel J Monocytes and tissue factor promote thrombosis in a murine model of oxygen deprivation. J Clin Invest (1997) 99(7):1729–38. 10.1172/JCI119337 PMC507994 9120018

[137] Keller TT van der Sluijs KF de Kruif MD Gerdes VE Meijers JC Florquin S Effects on coagulation and fibrinolysis induced by influenza in mice with a reduced capacity to generate activated protein C and a deficiency in plasminogen activator inhibitor type 1. Circ Res (2006) 99(11):1261–9. 10.1161/01.RES.0000250834.29108.1a 17068293

[138] Boffa MC Karmochkine M Thrombomodulin: an overview and potential implications in vascular disorders. Lupus (1998) 7(Suppl 2S120-5). 10.1177/096120339800700227 9814688

[139] van der Poll T Levi M Buller HR van Deventer SJ de Boer JP Hack CE Fibrinolytic response to tumor necrosis factor in healthy subjects. J Exp Med (1991) 174(3):729–32. 10.1084/jem.174.3.729 PMC2118940 1714936

[140] Kruithof EK Mestries JC Gascon MP Ythier A The coagulation and fibrinolytic responses of baboons after in vivo thrombin generation–effect of interleukin 6. Thromb Haemost (1997) 77(5):905–10. 10.1055/s-0038-1656076 9184401

[141] Wang ZF Su F Lin XJ Dai B Kong LF Zhao HW Serum D-dimer changes and prognostic implication in 2009 novel influenza A(H1N1). Thromb Res (2011) 127(3):198–201. 10.1016/j.thromres.2010.11.032 21216444

[142] Puntmann VO Carerj ML Wieters I Fahim M Arendt C Hoffmann J Outcomes of Cardiovascular Magnetic Resonance Imaging in Patients Recently Recovered From Coronavirus Disease 2019 (COVID-19). JAMA Cardiol (2020). 10.1001/jamacardio.2020.3557 PMC7385689 32730619

[143] Zhu L Liu L Zhang Y Pu L Liu J Li X High Level of Neutrophil Extracellular Traps Correlates With Poor Prognosis of Severe Influenza A Infection. J Infect Dis (2018) 217(3):428–37. 10.1093/infdis/jix475 29325098

[144] Moorthy AN Tan KB Wang S Narasaraju T Chow VT Effect of High-Fat Diet on the Formation of Pulmonary Neutrophil Extracellular Traps during Influenza Pneumonia in BALB/c Mice. Front Immunol (2016) 7:289. 10.3389/fimmu.2016.00289 27531997 PMC4969943

[145] Kotaka M Kitaura Y Deguchi H Kawamura K Experimental influenza A virus myocarditis in mice. Light and electron microscopic, virologic, and hemodynamic study. Am J Pathol (1990) 136(2):409–19. PMC1877391 2154929

[146] Pan HY Yamada H Chida J Wang S Yano M Up-regulation of ectopic trypsins in the myocardium by influenza A virus infection triggers acute myocarditis. Cardiovasc Res (2011) 89(3):595–603. 10.1093/cvr/cvq358 21084314 PMC3028976

[147] De Jesus NM Wang L Lai J Rigor RR Francis Stuart SD Rigor RR Antiarrhythmic effects of interleukin 1 inhibition after myocardial infarction. Heart Rhythm (2017) 14(5):727–36. 10.1016/j.hrthm.2017.01.027 PMC5403568 28111350

[148] De Jesus NM Wang L Herren AW Wang J Shenasa F Bers DM Atherosclerosis exacerbates arrhythmia following myocardial infarction: Role of myocardial inflammation. Heart Rhythm (2015) 12(1):169–78. 10.1016/j.hrthm.2014.10.007 PMC4277908 25304682

[149] Lazzerini PE Capecchi PL Laghi-Pasini F Systemic inflammation and arrhythmic risk: lessons from rheumatoid arthritis. Eur Heart J (2017) 38(22):1717–27. 10.1093/eurheartj/ehw208 27252448

[150] Monnerat G Alarcon ML Vasconcellos LR Hochman-Mendez C Brasil G Bassani RA Macrophage-dependent IL-1beta production induces cardiac arrhythmias in diabetic mice. Nat Commun (2016) 7:13344. 10.1038/ncomms13344 27882934 PMC5123037

[151] Madjid M Safavi-Naeini P Solomon SD Vardeny O Potential Effects of Coronaviruses on the Cardiovascular System: A Review. JAMA Cardiol (2020) 5(7):831–40. 10.1001/jamacardio.2020.1286 32219363

[152] Zhang Y Bauersachs J Langer HF Immune mechanisms in heart failure. Eur J Heart Fail (2017) 19(11):1379–89. 10.1002/ejhf.942 28891154

[153] Egorova EN Kalinkin MN Mazur ES [Immune mechanisms in pathogenesis of chronic heart failure]. Patol Fiziol Eksp Ter (2012) 2):56–61. 22708411

[154] Filgueiras-Rama D Vasilijevic J Jalife J Noujaim SN Alfonso JM Nicolas-Avila JA Human Influenza A virus causes myocardial and cardiac-specific conduction system infection associated with early inflammation and premature death. Cardiovasc Res (2020). 10.1093/cvr/cvaa117 PMC7898948 32346730

[155] Kenney AD McMichael TM Imas A Chesarino NM Zhang L Dorn LE IFITM3 protects the heart during influenza virus infection. Proc Natl Acad Sci U S A (2019) 116(37):18607–12. 10.1073/pnas.1900784116 PMC6744864 31451661

[156] Bangalore S Sharma A Slotwiner A Yatskar L Harari R Shah B ST-Segment Elevation in Patients with Covid-19 - A Case Series. N Engl J Med (2020) 382(25):2478–80. 10.1056/NEJMc2009020 PMC7182015 32302081

[157] Stefanini GG Montorfano M Trabattoni D Andreini D Ferrante G Ancona M ST-Elevation Myocardial Infarction in Patients With COVID-19: Clinical and Angiographic Outcomes. Circulation (2020) 141(25):2113–6. 10.1161/CIRCULATIONAHA.120.047525 PMC7302062 32352306

[158] Hamadeh A Aldujeli A Briedis K Tecson KM Sanz-Sanchez J Al Dujeili M Characteristics and Outcomes in Patients Presenting With COVID-19 and ST-Segment Elevation Myocardial Infarction. Am J Cardiol (2020), 1311–6. 10.1016/j.amjcard.2020.06.063 PMC7333635 32732010

[159] Ho JS Sia CH Chan MY Lin W Wong RC Coronavirus-induced myocarditis: A meta-summary of cases. Heart Lung (2020) 49(6):681–5. 10.1016/j.hrtlng.2020.08.013 PMC7440036 32861884

[160] Davidovic G Simovic S Mitrovic S Iric-Cupic V Miloradovic V Fulminant myocarditis as a primary manifestation of H1N1 infection: A first reported case from Serbia. Hellenic J Cardiol (2016) 57(3):181–4. 10.1016/j.hjc.2015.06.001 27725100

[161] Cabral M Brito MJ Conde M Oliveira M Ferreira GC Fulminant myocarditis associated with pandemic H1N1 influenza Avirus. Rev Port Cardiol (2012) 31(7-8):517–1159, 20. 10.1016/j.repc.2011.11.012 22704822

[162] Khouzam RN Parizianu C Hafiz AM Chawla S Schwartz R Fulminant myocarditis associated with novel H1N1 influenza A. Heart Lung (2011) 40(6):566–8. 10.1016/j.hrtlng.2011.01.004 21411147

[163] Komai T Nakazawa G Asai S Ikari Y Fatal fulminant myocarditis associated with novel influenza A (H1N1) infection. Eur Heart J (2011) 32(3):283. 10.1093/eurheartj/ehq359 20861138

[164] Gross ER Gander JW Reichstein A Cowles RA Stolar CJ Middlesworth W Fulminant pH1N1-09 influenza-associated myocarditis in pediatric patients. Pediatr Crit Care Med (2011) 12(2):e99–e101. 10.1097/PCC.0b013e3181e28887 20601924 PMC4425292

[165] Cuomo V Esposito R Santoro C Fulminant myocarditis in the time of coronavirus. Eur Heart J (2020) 41(22):2121. 10.1093/eurheartj/ehaa354 32338735 PMC7197560

[166] Joob B Wiwanitkit V Fulminant myocarditis and COVID-19. Rev Esp Cardiol (Engl Ed) (2020). 10.1016/j.rec.2020.05.006 PMC7236670 32517999

[167] Craver R Huber S Sandomirsky M McKenna D Schieffelin L Finger D Fatal Eosinophilic Myocarditis in a Healthy 17-Year-Old Male with Severe Acute Respiratory Syndrome Coronavirus 2 (SARS-CoV-2c). Fetal Pediatr Pathol (2020) 1–6. 10.1080/15513815.2020.1761491 PMC7232882 32401577

[168] Zeng JH Liu YX Yuan J Wang FX Wu WB Li JX First case of COVID-19 complicated with fulminant myocarditis: a case report and insights. Infection (2020) 48(5):773–7. 10.1007/s15010-020-01424-5 PMC7146072 32277408

[169] Riphagen S Gomez X Gonzalez-Martinez C Wilkinson N Theocharis P Hyperinflammatory shock in children during COVID-19 pandemic. Lancet (2020) 395(10237):1607–8. 10.1016/S0140-6736(20)31094-1 PMC7204765 32386565

[170] Jones VG Mills M Suarez D Hogan CA Yeh D COVID-19 and Kawasaki Disease: Novel Virus and Novel Case. Hosp Pediatr (2020) 10(6):537–40. 10.1542/hpeds.2020-0123 32265235

[171] Toubiana J Poirault C Corsia A Bajolle F Fourgeaud J Angoulvant F Kawasaki-like multisystem inflammatory syndrome in children during the covid-19 pandemic in Paris, France: prospective observational study. BMJ (2020) 369:m2094. 10.1136/bmj.m2094 32493739 PMC7500538

[172] Whittaker E Bamford A Kenny J Kaforou M Jones CE Shah P Clinical Characteristics of 58 Children With a Pediatric Inflammatory Multisystem Syndrome Temporally Associated With SARS-CoV-2. JAMA (2020) 324(3):259–69. 10.1001/jama.2020.10369 PMC7281356 32511692

[173] Mubbasheer Ahmed SA Moreira A Zoretic S Martinez J Chorath K Acosta S Multisystem inflammatory syndrome in children: A systematic review. Lancet (2020) 26:100527. 10.1016/j.eclinm.2020.100527 PMC7473262 32923992

[174] Guo T Fan Y Chen M Wu X Zhang L He T Cardiovascular Implications of Fatal Outcomes of Patients With Coronavirus Disease 2019 (COVID-19). JAMA Cardiol (2020) 5(7):811–8. 10.1001/jamacardio.2020.1017 PMC7101506 32219356

[175] Goyal P Choi JJ Pinheiro LC Schenck EJ Chen R Jabri A Clinical Characteristics of Covid-19 in New York City. N Engl J Med (2020) 382(24):2372–4. 10.1056/NEJMc2010419 PMC7182018 32302078

[176] Kochav SM Coromilas E Nalbandian A Ranard LS Gupta A Chung MK Cardiac Arrhythmias in COVID-19 Infection. Circ Arrhythm Electrophysiol (2020) 13(6):e008719. 10.1161/CIRCEP.120.008719 32434385 PMC7299099

[177] Elsaid O McCullough PA Tecson KM Williams RS Yoon A Ventricular Fibrillation Storm in Coronavirus 2019. Am J Cardiol (2020). 10.1016/j.amjcard.2020.08.033 PMC7455792 32871109

[178] Wang D Hu B Hu C Zhu F Liu X Zhang J Clinical Characteristics of 138 Hospitalized Patients With 2019 Novel Coronavirus-Infected Pneumonia in Wuhan, China. JAMA (2020) 323(11):1061–9. 10.1001/jama.2020.1585 PMC7042881 32031570

[179] Abrams MP Coromilas EJ Wan EY Rubin GA Garan H Dizon JM Malignant Ventricular Arrhythmias in Patients with Severe Acute Respiratory Distress Syndrome Due to COVID-19 without Significant Structural Heart Disease. HeartRhythm Case Rep (2020). 10.1016/j.hrcr.2020.08.017 PMC7446721 32864335

[180] Rivers J Ihle JF COVID-19 social isolation-induced takotsubo cardiomyopathy. Med J Aust (2020). 10.5694/mja2.50770 32909275

[181] Sattar Y Connerney M Ullah W Philippou A Slack D McCarthy B COVID-19 Presenting as Takotsubo Cardiomyopathy Complicated with AtrialFibrillation. Int J Cardiol Heart Vasc (2020) 29 100580. 10.1016/j.ijcha.2020.100580 32685662 PMC7348613

[182] Tsao CW Strom JB Chang JD Manning WJ COVID-19-Associated Stress (Takotsubo) Cardiomyopathy. Circ Cardiovasc Imaging (2020) 13(7):e011222. 10.1161/CIRCIMAGING.120.011222 32673494 PMC7398589

[183] Giustino G Croft LB Oates CP Rahman K Lerakis S Reddy VY Takotsubo Cardiomyopathy in COVID-19. J Am Coll Cardiol (2020) 76(5):628–9. 10.1016/j.jacc.2020.05.068 PMC7279731 32517962

[184] Nguyen D Nguyen T De Bels D Castro Rodriguez J A case of Takotsubo cardiomyopathy with COVID 19. Eur Heart J Cardiovasc Imaging (2020) 21(9):1052. 10.1093/ehjci/jeaa152 32395765 PMC7239208

[185] Chadha S ‘COVID-19 pandemic’ anxiety-induced Takotsubo cardiomyopathy. QJM (2020) 113(7):488–90. 10.1093/qjmed/hcaa135 PMC7188117 32311043

[186] Nakamura M Nakagaito M Hori M Ueno H Kinugawa K A case of Takotsubo cardiomyopathy with cardiogenic shock after influenza infection successfully recovered by IMPELLA support. J Artif Organs (2019) 22(4):330–3. 10.1007/s10047-019-01112-8 31228028

[187] Elikowski W Malek-Elikowska M Lisiecka M Trypuc Z Mozer-Lisewska I Takotsubo cardiomyopathy triggered by influenza B. Pol Merkur Lekarski (2018) 45(266):67–70. 30240371

[188] Buzon J Roignot O Lemoine S Perez P Kimmoun A Levy B Takotsubo Cardiomyopathy Triggered by Influenza A Virus. Intern Med (2015) 54(16):2017–9. 10.2169/internalmedicine.54.3606 26278294

[189] Singh K Marinelli T Horowitz JD Takotsubo cardiomyopathy after anti-influenza vaccination: catecholaminergic effects of immune system. Am J Emerg Med (2013) 31(11):1627 e1–4. 10.1016/j.ajem.2013.06.039 23891597

[190] Shi S Qin M Shen B Cai Y Liu T Yang F Association of Cardiac Injury With Mortality in Hospitalized Patients With COVID-19 in Wuhan, China. JAMA Cardiol (2020) 5(7):802–10. 10.1001/jamacardio.2020.0950 PMC7097841 32211816

[191] Cao J Zheng Y Luo Z Mei Z Yao Y Liu Z Myocardial injury and COVID-19: Serum hs-cTnI level in risk stratification and the prediction of 30-day fatality in COVID-19 patients with no prior cardiovascular disease. Theranostics (2020) 10(21):9663–73. 10.7150/thno.47980 PMC7449913 32863952

[192] Klok FA Kruip M van der Meer NJM Arbous MS Gommers D Kant KM Confirmation of the high cumulative incidence of thrombotic complications in critically ill ICU patients with COVID-19: An updated analysis. Thromb Res (2020) 191148–50. 10.1016/j.thromres.2020.04.041 PMC7192101 32381264

[193] Mazo J Singh S Khan Z Foster A Komarnitsky E More than Just Pneumonia: Acute Pulmonary Embolism in Two Middle-Aged Patients with COVID-19. Case Rep Med (2020) 2020 4812036. 10.1155/2020/4812036 32774385 PMC7396098

[194] Chen J Wang X Zhang S Lin B Wu X Wang Y Characteristics of Acute Pulmonary Embolism in Patients With COVID-19 AssociatedPneumonia From the City of Wuhan. Clin Appl Thromb Hemost (2020) 26:1–8. 10.1177/1076029620936772 PMC7391435 32726134

[195] Gervaise A Bouzad C Peroux E Helissey C Acute pulmonary embolism in non-hospitalized COVID-19 patients referred to CTPA by emergency department. Eur Radiol (2020) 30(11):6170–7. 10.1007/s00330-020-06977-5 PMC7280685 32518989

[196] Faggiano P Bonelli A Paris S Milesi G Bisegna S Bernardi N Acute pulmonary embolism in COVID-19 disease: Preliminary report on seven patients. Int J Cardiol (2020) 313:129–31. 10.1016/j.ijcard.2020.04.028 PMC7250100 32471650

[197] Roncon L Zuin M Zonzin P Fibrinolysis in COVID-19 patients with hemodynamic unstable acute pulmonary embolism: yes or no? J Thromb Thrombolysis (2020) 50(1):221–2. 10.1007/s11239-020-02131-6 PMC7213545 32394238

[198] Leonard-Lorant I Delabranche X Severac F Helms J Pauzet C Collange O Acute Pulmonary Embolism in Patients with COVID-19 at CT Angiography and Relationship to d-Dimer Levels. Radiology (2020) 296(3):E189–E91. 10.1148/radiol.2020201561 PMC7233397 32324102

[199] Cui S Chen S Li X Liu S Wang F Prevalence of venous thromboembolism in patients with severe novel coronavirus pneumonia. J Thromb Haemost (2020) 18(6):1421–4. 10.1111/jth.14830 PMC7262324 32271988

[200] Wichmann D Sperhake JP Lutgehetmann M Steurer S Edler C Heinemann A Autopsy Findings and Venous Thromboembolism in Patients With COVID-19: A Prospective Cohort Study. Ann Intern Med (2020) 173(4):268–77. 10.7326/M20-2003 PMC7240772 32374815

[201] Tang N Li D Wang X Sun Z Abnormal coagulation parameters are associated with poor prognosis in patients with novel coronavirus pneumonia. J Thromb Haemost (2020) 18(4):844–7. 10.1111/jth.14768 PMC7166509 32073213

[202] Llitjos JF Leclerc M Chochois C Monsallier JM Ramakers M Auvray M High incidence of venous thromboembolic events in anticoagulated severe COVID-19 patients. J Thromb Haemost (2020) 18(7):1743–6. 10.1111/jth.14869 PMC7264774 32320517

[203] Carsana L Sonzogni A Nasr A Rossi RS Pellegrinelli A Zerbi P Pulmonary post-mortem findings in a series of COVID-19 cases from northern Italy: a two-centre descriptive study. Lancet Infect Dis (2020) 20(10):1135–40. 10.1016/S1473-3099(20)30434-5 PMC7279758 32526193

[204] Bunce PE High SM Nadjafi M Stanley K Liles WC Christian MD Pandemic H1N1 influenza infection and vascular thrombosis. Clin Infect Dis (2011) 52(2):e14–7. 10.1093/cid/ciq125 21288835

[205] Ackermann M Verleden SE Kuehnel M Haverich A Welte T Haverich A Pulmonary Vascular Endothelialitis, Thrombosis, and Angiogenesis in Covid-19. N Engl J Med (2020) 383(2):120–8. 10.1056/NEJMoa2015432 PMC7412750 32437596

[206] Zou X Chen K Zou J Han P Hao J Han Z Single-cell RNA-seq data analysis on the receptor ACE2 expression reveals the potential risk of different human organs vulnerable to 2019-nCoV infection. Front Med (2020) 14(2):185–92. 10.1007/s11684-020-0754-0 PMC7088738 32170560

[207] Hamming I Timens W Bulthuis ML Lely AT Navis G van Goor H Tissue distribution of ACE2 protein, the functional receptor for SARS coronavirus. A first step in understanding SARS pathogenesis. J Pathol (2004) 203(2):631–7. 10.1002/path.1570 PMC7167720 15141377

[208] Varga Z Flammer AJ Steiger P Haberecker M Andermatt R Zinkernagel AS Endothelial cell infection and endotheliitis in COVID-19. Lancet (2020) 395(10234):1417–8. 10.1016/S0140-6736(20)30937-5 PMC7172722 32325026

[209] Lindner D Fitzek A Brauninger H Aleshcheva G Edler C Meissner K Association of Cardiac Infection With SARS-CoV-2 in Confirmed COVID-19 Autopsy Cases. JAMA Cardiol (2020). 10.1001/jamacardio.2020.3551 PMC7385672 32730555

[210] Dolhnikoff M Ferreira Ferranti J de Almeida Monteiro RA Duarte-Neto AN Soares Gomes-Gouvea M Viu Degaspare N SARS-CoV-2 in cardiac tissue of a child with COVID-19-related multisystem inflammatory syndrome. Lancet Child Adolesc Health (2020). 10.1016/S2352-4642(20)30257-1 PMC7440866 32828177

[211] Sharma A Garcia G Jr. Wang Y Plummer JT Morizono K Morizono K Human iPSC-Derived Cardiomyocytes Are Susceptible to SARS-CoV-2 Infection. Cell Rep Med (2020) 1(4):100052. 10.1016/j.xcrm.2020.100052 32835305 PMC7323681

[212] Blanco-Melo D Nilsson-Payant BE Liu WC Uhl S Hoagland D Moller R Imbalanced Host Response to SARS-CoV-2 Drives Development of COVID-19. Cell (2020) 181(5):1036-45. 10.1016/j.cell.2020.04.026 PMC7227586 32416070

[213] Channappanavar R Fehr AR Vijay R Mack M Zhao J Meyerholz DK Dysregulated Type I Interferon and Inflammatory Monocyte-Macrophage Responses Cause Lethal Pneumonia in SARS-CoV-Infected Mice. Cell Host Microbe (2016) 19(2):181–93. 10.1016/j.chom.2016.01.007 PMC4752723 26867177

[214] Hadjadj J Yatim N Barnabei L Corneau A Boussier J Smith N Impaired type I interferon activity and inflammatory responses in severe COVID-19 patients. Science (2020) 369(6504):718–24. 10.1126/science.abc6027 PMC7402632 32661059

[215] Qin C Zhou L Hu Z Zhang S Yang S Tao Y Dysregulation of immune response in patients with COVID-19 in Wuhan, China. Clin Infect Dis (2020) 71(15):762–8. 10.1093/cid/ciaa248 PMC7108125 32161940

[216] Chen G Wu D Guo W Cao Y Huang D Wang H Clinical and immunological features of severe and moderate coronavirus disease 2019. J Clin Invest (2020) 130(5):2620–9. 10.1172/JCI137244 PMC7190990 32217835

[217] Agewall S Beltrame JF Reynolds HR Niessner A Rosano G Caforio AL ESC working group position paper on myocardial infarction with non-obstructive coronary arteries. Eur Heart J (2017) 38(3):143–53. 10.1093/eurheartj/ehw149 28158518

[218] Hottz ED Azevedo-Quintanilha IG Palhinha L Teixeira L Barreto EA Pao CRR Platelet activation and platelet-monocyte aggregates formation trigger tissue factor expression in severe COVID-19 patients. Blood (2020). 10.1182/blood.2020007252 PMC7483437 32678428

[219] He M He X Xie Q Chen F He S Angiotensin II induces the expression of tissue factor and its mechanism in human monocytes. Thromb Res (2006) 117(5):579–90. 10.1016/j.thromres.2005.04.033 15953627

[220] Goshua G Pine AB Meizlish ML Chang CH Zhang H Bahel P Endotheliopathy in COVID-19-associated coagulopathy: evidence from a single-centre, cross-sectional study. Lancet Haematol (2020) 7(8):e575–e82. 10.1016/S2352-3026(20)30216-7 PMC7326446 32619411

[221] Akiyama R Komori I Hiramoto R Isonishi A Matsumoto M Fujimura Y H1N1 influenza (swine flu)-associated thrombotic microangiopathy with a markedly high plasma ratio of von Willebrand factor to ADAMTS13. Intern Med (2011) 50(6):643–7. 10.2169/internalmedicine.50.4620 21422695

[222] van Wissen M Keller TT van Gorp EC Gerdes VE Meijers JC van Doornum GJ Acute respiratory tract infection leads to procoagulant changes in human subjects. J Thromb Haemost (2011) 9(7):1432–4. 10.1111/j.1538-7836.2011.04340.x PMC7166935 21605331

[223] Tong M Jiang Y Xia D Xiong Y Zheng Q Chen F Elevated Expression of Serum Endothelial Cell Adhesion Molecules in COVID-19 Patients. J Infect Dis (2020) 222(6):894–8. 10.1093/infdis/jiaa349 PMC7337874 32582936

[224] Nicolai L Leunig A Brambs S Kaiser R Weinberger T Weigand R Immunothrombotic Dysregulation in COVID-19 Pneumonia is Associated with Respiratory Failure and Coagulopathy. Circulation (2020) 142(12):1176–89. 10.1161/CIRCULATIONAHA.120.048488 PMC7497892 32755393

